# Plant root associated chitinases: structures and functions

**DOI:** 10.3389/fpls.2024.1344142

**Published:** 2024-02-01

**Authors:** Samuel O. Shobade, Olga A. Zabotina, Marit Nilsen-Hamilton

**Affiliations:** ^1^ Ames National Laboratory, U. S. Department of Energy, Ames, IA, United States; ^2^ Roy J. Carver Department of Biochemistry, Biophysics and Molecular Biology, Iowa State University, Ames, IA, United States

**Keywords:** chitinase, hydrolases, chitin-binding domain, C-terminal domain, anti-fungal activity, rhizosphere

## Abstract

Chitinases degrade chitin, a linear homopolymer of β-1,4-linked N-acetyl-D-glucosamine (GlcNAc) residues found in the cell walls of fungi and the exoskeletons of arthropods. They are secreted by the roots into the rhizosphere, a complex and dynamic environment where intense nutrient exchange occurs between plants and microbes. Here we modeled, expressed, purified, and characterized *Zea mays* and *Oryza sativa* root chitinases, and the chitinase of a symbiotic bacterium, *Chitinophaga oryzae* 1303 for their activities with chitin, di-, tri-, and tetra-saccharides and *Aspergillus niger*, with the goal of determining their role(s) in the rhizosphere and better understanding the molecular mechanisms underlying plant-microbe interactions. We show that *Zea mays* basic endochitinase (*Zm*Chi19A) and *Oryza sativa* chitinase (*Os*Chi19A) are from the GH19 chitinase family. The *Chitinophaga oryzae* 1303 chitinase (*Csp*Ch18A) belongs to the GH18 family. The three enzymes have similar apparent *K*_M_ values of (20-40 µM) for the substrate 4-MU-GlcNAc_3_. They vary in their pH and temperature optima with *Os*Chi19A activity optimal between pH 5–7 and 30–40°C while *Zm*Chi19A and *Csp*Ch18A activities were optimal at pH 7-9 and 50–60°C. Modeling and site-directed mutation of *Zm*Chi19A identified the catalytic cleft and the active residues E147 and E169 strategically positioned at ~8.6Å from each other in the folded protein. Cleavage of 4-MU-GlcNAc_3_ was unaffected by the absence of the CBD but diminished in the absence of the flexible C-terminal domain. However, unlike for the soluble substrate, the CBD and the newly identified flexible C-terminal domain were vital for inhibiting *Aspergillus niger* growth. The results are consistent with the involvement of the plant chitinases in defense against pathogens like fungi that have chitin exoskeletons. In summary, we have characterized the functional features and structural domains necessary for the activity of two plant root chitinases that are believed to be involved in plant defense and a bacterial chitinase that, along with the plant chitinases, may participate in nutrient recycling in the rhizosphere.

## 1 Introduction

Agricultural crops have great economic importance and with the worldwide population increase, the current crop production rate is not sufficient to feed the future human population (Pingali, 2012; Kc et al., 2018). Crops suffer attack by pathogens including fungi, bacteria, and viruses with fungi alone causing 26 – 30% of the yield losses for crops like wheat, sugar beet and cotton (Roy et al., 2021) and 35% to 40% of the damage in maize, potato and rice (Kc et al., 2018). Maize (*Zea mays*) and rice (*Oryza sativa*) are among the most important food crops globally, and their growth and productivity are greatly influenced by the rhizosphere (Roy et al., 2021; Yim et al., 2022; USDA, 2023). Thus, a large increase in crop yield with the consequent alleviation of food insecurity for millions of people can be achieved by successfully addressing the challenges of plant stress such as induced by fungal infection (Pingali, 2012; Haldar and Sengupta, 2015; Rizzo et al., 2021).

Chitinases are hydrolytic enzymes that degrade chitin, a straight-chain homopolymer of β-1,4-linked N-acetyl-D-glucosamine (GlcNAc) units found in arthropod exoskeletons and some fungi cell walls (Martínez-Caballero et al., 2014; Horiuchi et al., 2016; Oyeleye and Normi, 2018; Wang et al., 2019). By breaking down chitin, chitinases inhibit fungal growth and release essential nutrients that plants can use for growth and development. Chitinases are expressed by a variety of organisms, including fungi, bacteria, archaea, viruses, animals, and plants. They are classified into the GH18, GH19 and GH20 families based on the CAZy database (Oyeleye and Normi, 2018). GH18 chitinases are widely distributed in eukaryotes and prokaryotes (Ju et al., 2016; Wang et al., 2019; Renaud et al., 2023) while GH19 chitinases are mostly found in plants.

Plant roots secrete chitinases into the rhizosphere (Haldar and Sengupta, 2015; Wang et al., 2019; Yim et al., 2022), which is a complex and dynamic environment where intense nutrient exchange occurs between plants and microbes with important consequences for plant growth, health, and productivity (Haldar and Sengupta, 2015). As part of the plant’s defense response, chitinases can lyse pathogens directly or indirectly by weakening their cell walls. The expression of root chitinases is also influenced by rhizosphere microbes such as fungi and bacteria, which can activate or suppress the synthesis of these enzymes. This connection is bidirectional, with root chitinases influencing the quantity and diversity of rhizosphere microorganisms and rhizosphere bacteria influencing the expression and activity of root chitinases (Haldar and Sengupta, 2015; Roy et al., 2021; Yim et al., 2022).

To better understand the biochemical and molecular features of chitinases that function in the soil, we have identified chitinases that are expressed by *Zea mays* and *Oryza sativa* roots (Vega-Arreguín et al., 2009; The, 2018) and found in their root exudates (Alexandrov et al., 2009; Ma et al., 2010) These plant chitinases and a chitinase from the genus *Chitinophaga*, which includes a number of soil-dwelling bacterial species, were expressed, and characterized. Phylogenetic analysis and molecular modeling identified the class and structure of each. Although the GH19 plant chitinases possess a chitin binding domain, which is absent from the bacterial chitinase, the three chitinases have similar activity on colloidal chitin and similar kinetic parameters assessed by a trimeric saccharide substrate. pH and temperature optima and stabilities are like those reported for other chitinases. Studies of truncated versions of the *Zea mays* chitinase identified a C-terminal domain which, like the chitin binding domain, is not required for cleaving short oligosaccharides, but is required for cleaving colloidal chitin and for attacking the fungal cell wall. These chitinases have potential for industrial application and will provide meaningful biomarkers for tracking plant root activity *in situ* in response to stresses such as fungal invasion.

## 2 Materials and methods

### 2.1 Reagents

The fluorogenic soluble substrates used for enzymatic assays: 4-Methylumbelliferyl β-D-N,N′-diacetylchitobioside (Cat#53643-12-2), 4-Methylumbelliferyl β-D-N,N′,N′′-triacetylchitotrioside (Cat#M5639-5MG) and 4-Methylumbelliferyl β-D-N,N’,N”,N’’’-Tetraacetylchitotetraoside (Cat#53643-14-4) were purchased from Cayman Chemicals (Ann Arbor, MA, USA), Sigma Aldrich (St. Louis, MO, USA) and Toronto Research Chemicals (Toronto, ON, Canada) respectively in powder form and dissolved in appropriate solvents to make stock solutions according to the manufacturer’s instructions. Colloidal chitin for chitinase activity comparisons was prepared from chitin (from shells of lobster, crab, or shrimp) (Cat#1398-61-4, J61206) by dissolving 5 g of powdered chitin in 250 mL of cold concentrated HCl or 85% phosphoric acid and allowed to rest at 4 °C for 24 h. The resulting suspension was passed through layers of cheese cloth to remove chunks, then placed on layers of filter papers and washed with cold tap water until pH of the rinse was ~7.0 (tested with pH paper). The paste was then stored at 4 °C to be weighed and resuspended in desired buffers when needed. DNS (3,5 Dinitrosalicylic acid, 98%, Cat#609-99-4), used for reaction termination to quantify the reducing sugar released from chitinase activity reactions, was purchased from Fisher Scientific (Waltham, MA, USA). TALON Metal Affinity Resin (Cat#635502) purchased from TakaraBio (San Jose, CA, USA) and Ni-NTA affinity Resin (Cat#R90115) purchased from Thermofisher Scientific (Waltham, MA, USA) were used for protein purifications. Other chemical reagents were of analytical grade or higher purity and were obtained from Sigma-Aldrich (St. Louis, MO, USA). Single and multiple site-directed mutagenesis of the chitinases were conducted with the GeneArt^®^ Site-Directed Mutagenesis PLUS Kit (Cat #A14604, Thermofisher Scientific, Waltham, MA) using the AccuPrime™ Pfx DNA Polymerase (Cat#12344-024, Thermofisher Scientific, Waltham, MA). The protein standards used to determine the protein molecular weights were the broad range color prestained protein standard (10-250 kDa) (Cat#P7719S) from New England Biolabs (NEB) (Ipswich, MA) and the broad range spectra multicolor (Product# 26634) purchased from Thermofisher Scientific (Waltham, MA). Oligonucleotides were purchased from Integrated DNA Technologies (IDT, Coralville, IA), The sequences of all oligonucleotides used in this study are listed in **Supplementary Table S1**.

### 2.2 Identification of chitinases secreted into the rhizosphere

Candidate maize (*Zea mays* L.) chitinase (Basic Endochitinase A, *Zm*Chi19A) was selected from stress response proteins identified to be secreted into the root mucilage (Ma et al., 2010). The nucleotide sequences of maize *Zm*Chi19A, rice (*Oryza sativa*) root chitinase, *Os*Chi19A, and symbiotic bacteria *Chitinophaga oryzae* 1303 chitinase (*Csp*Ch18A) were retrieved from the nucleotide database of the National Center for Biotechnology Information (NCBI, https://www.ncbi.nlm.nih.gov/) (Alexandrov et al., 2009).

### 2.3 Evolutionary relationship, sequence alignment, and glycosylation sites

Identified gene sequences were translated using the Expasy translate tool (https://web.expasy.org/translate/) while the protein parameters were obtained using the Expasy ProtParam tool (https://web.expasy.org/protparam/) (Gasteiger et al., 2007). Phylogenic analysis was carried out to determine the evolutionary relationship between the two plant chitinases as well as the bacterial chitinase (http://www.phylogeny.fr/) (Dereeper et al., 2008). Multiple sequence alignment was performed using the ClustalW algorithm (https://www.ebi.ac.uk/Tools/msa/clustalo/) (Sievers and Higgins, 2014), while conserved motifs of the protein sequences were analyzed using (https://www.ncbi.nlm.nih.gov/Structure/cdd/wrpsb.cgi ) (Lu et al., 2020). The prediction of the signal peptide sequence was performed using the signal-5.0 application server at https://services.healthtech.dtu.dk/services/SignalP-5.0/. To predict N- and O-glycosylation sites, the servers NetNGlyc 1.0 (https://services.healthtech.dtu.dk/service.php?NetNGlyc-1.0) (Gupta and Brunak, 2001) and NetOGlyc 4.0 (https://services.healthtech.dtu.dk/service.php?NetOGlyc-4.0) (Steentoft et al., 2013) were used respectively.

### 2.4 Homology models, structural alignment, and surface charge distribution

Prediction of protein structures and mobility were done using AlphaFold2 (Jumper et al., 2021). The surface charge distribution and structural alignment of the modeled protein structures was determined using the Poisson Boltzmann tool at https://server.poissonboltzmann.org/ (Jurrus et al., 2018) and https://zhanglab.ccmb.med.umich.edu/TM-align/ (Zhang and Skolnick, 2005) respectively. Structure visualization, analysis, and representations were done using PyMOL and ChimeraX softwares (Pettersen et al., 2004).

### 2.5 Molecular docking

To predict the catalytic residues, docking of the structures with a chitin substrate was done with HADDOCK, SWISSDOCK and CBDOCK-2 (Grosdidier et al., 2011; Honorato et al., 2021; Liu et al., 2022). The chitin substrate was obtained from the PDB structure 6BN0 (Hurlburt et al., 2018).

### 2.6 Cloning

The *Zm*Chi19A coding sequence was amplified from the maize root cDNA and cloned into the pET28b vector for *E. coli* expression. The pET28b expression vector had been modified to incorporate an N-terminal 10x-His and SUMO solubility tag. Overhangs of the forward and reverse primers were designed to contain the BamHI and HindIII restriction sites respectively. Gene block fragments of *Os*Chi19A and *Csp*Ch18A were cloned into the pET28a vector for *E. coli* expression (Horiuchi et al., 2016). Overhangs of the forward primers were designed to contain the XbaI restriction site, and the reverse primers were designed to contain the XhoI restriction site. Gene sequence of *Os*Chi19A was also codon modified for optimal expression in *E. coli*. All forward primers were designed to produce the Tobacco Etch Virus (TEV) protease site (ENLYFQG) at the N-terminus to provide options of cutting off the tags after expression and purification. Primers were also designed to truncate the signal peptides located on the N-terminal of the gene sequences. The *Zm*Chi19A(E147A, E169A) mutant was prepared using a designed Gene Block fragment from Integrated DNA Technologies (IDT, Coralville, IA), while truncations of the CBD (*Zm*Chi19AΔCBD) and flexible C-terminal (*Zm*Chi19A_A328*) were achieved by PCR amplification of the cDNA using primers designed to make the truncations, PCR amplification of the truncated cDNA, followed by subsequent and insertion between the Xho1 and Xba1 sites of the expression vector. *Csp*Ch18A(D161A, E163A) mutant was prepared by site-directed mutagenesis (Wang et al., 2019) using the GeneArt^®^ Site-Directed Mutagenesis PLUS Kit (Cat #A14604, Thermofisher Scientific, Waltham, MA) and AccuPrime™ Pfx DNA Polymerase (Cat#12344-024, Thermofisher Scientific, Waltham, MA). The numbers assigned to the mutant amino acid residues correspond to the positions of the amino acids in the complete translated protein, starting with methionine and including the signal sequence. Restriction enzymes used were purchased from New England Biolabs (NEB, Ipswich, MA). T4 DNA Ligase was obtained from Promega (Cat#C126A, Madison, WI). Chemically competent cells used for cloning (*E. coli* 10G & DH5α) were purchased from Lucigen (Cat. # 60107-1, Middleton, WI) and Thermofisher Scientific (Cat#12297-016) respectively. For protein expression, plasmids were retransformed into OverExpress chemically competent cells C43(DE3) (Cat. # 60446-1, Lucigen, Middleton, WI) and Rosetta-gamiTM^2^(DE3)pLysS Chemically Competent Cells were from Novagen (Cat#71432-3, Sigma-Aldrich, St. Louis, MO). All the recombinant plasmids were verified by Sanger sequencing at the Iowa State University (ISU) DNA facility. Gene sequences and primers used for cloning can be found in the **Supplementary Data**.

### 2.7 Protein expression and purification

Competent cells harboring the expression plasmids were grown at 37°C with shaking at 250 rpm in 1000 ml of Luria-Bertani broth. When the cell culture reached an OD_600_ of 0.4, the temperature was lowered to 18°C, incubated for 15 mins and protein expression was induced by adding IPTG to a final concentration of 1.0 mM. Cells expressing all but *Zm*Chi19AΔCBD were incubated for 18 h at 18°C and *Zm*Chi19AΔCBD-transformed cells were incubated at 10 °C for 72 h. Induced cells were harvested by centrifugation, resuspended in lysis buffer (12.5 mL of 25 mM Tris-HCl pH 7.4, 300 mM NaCl, 0.1 mM EDTA) (Deshmukh et al., 2023) and rapidly frozen in liquid nitrogen. Cells were lysed by thawing and incubating for 30 min with 1 mg/mL of lysozyme and then sonicated for 15 s a total of five times. Solubilized proteins were collected by centrifugation at 15,000 *x g* for 30 min to obtain the crude lysates (Horiuchi et al., 2016; Singappuli-Arachchige et al., 2022). *Os*Chi19A was further purified from inclusion bodies in the pellet and refolded using a refolding buffer (50 mM HEPES, 0.4 M L-Arginine, 6.3 mM GSH, 3.7 mM GSSG, 2 mM EDTA, pH 7.0). Crude lysate was loaded onto a TALON or Ni-NTA column with a lysate:resin ratio of 10:1 (v/v). The affinity resin was incubated on a shaker for 18 h at 4°C. Unbound proteins were removed as a flow-through fraction and the resin was washed five times with washing buffer (50 mM Tris-HCl (pH 7.4), 150 mM NaCl, and 20 mM imidazole). The proteins of interest were eluted in fractions with volumes of 1 mL using elution buffer (50 mM Tris-HCl pH 7.4, 150 mM NaCl, and 250 mM imidazole) (Wang et al., 2019) Dialysis into storage buffer (50mM Tris-HCl pH 7.4, 150 mM NaCl) was performed using a dialysis bag to eliminate imidazole. The proteins were concentrated from the elution buffer using Amicon Ultra centrifuge filter units (30,000 Da cutoff, Millipore, Burlington, MA). Glycerol was added to the purified protein to a final concentration of 25% and proteins were stored at -80°C. The concentrations of proteins were estimated from absorption at 280 nm determined by a NanoDrop spectrophotometer (#ND-1000, Thermo Scientific/Gibco, Waltham, MA) and using an extinction coefficient calculate for each protein based on its amino acid content and the presence of free SH groups (Gasteiger et al., 2007) and by the Bradford assay (Quick Start Bradford Dye reagent 1X, Cat#500-0205, Bio-Rad, Hercules, CA) according to the manufacturer’s instructions. Reducing and non-reducing SDS polyacrylamide gel electrophoresis (SDS-PAGE) for proteins were performed as previously described using 10 or 12% acrylamide gels (Nilsen-Hamilton et al., 1980). The gels were run for 2 h at constant current of 25 mA (Nilsen-Hamilton and Hamilton, 1987). All purified recombinant proteins were verified by Liquid Chromatography Mass Spectrometry (LC-MS) at the Iowa State University (ISU) Protein facility. Approximations for the purities of proteins used in this study are shown in **Supplementary Table S2**.

### 2.8 Size exclusion chromatography

Size exclusion chromatography was performed 4 °C in an AKTA FPLC system with a pre- packed Superose 12 10/300GL (separation range: 1 kDa to 300 kDa; GE Healthcare, Cat#17517301, Waukesha, WI) with a flow rate of 0.2 mL/min. The inner dimensions of the column were 10x300-310 mm with a bed volume of 24 mL. Prior to being loaded on the column, samples were dialyzed against the column buffer (20 mM Tris, 100 mM KCl, pH 7.9) then centrifuged at 15,900 RCF at 4 °C for 30 min.

### 2.9 CD spectroscopy

The overall secondary structures of purified proteins were investigated at 25 °C using a J-810 circular dichroism (CD) spectropolarimeter (Jasco, Hachioji, Tokyo, Japan). CD spectra were collected from 170 to 270 nm at a scanning rate of 200 nm/min with a path length of 0.1 cm (Figueroa et al., 2016; Giudice et al., 2017).

### 2.10 Enzyme activity assay

Enzyme kinetics assays were carried out with the fluorogenic substrate 4-Methylumbelliferyl β-DN,N′,N′′-triacetylchitotrioside [4-MU-(GlcNAc)_3_] substrate at concentrations from 5 to 100 µM. All enzymes used for determining the kinetic parameters were FPLC-purified. The optimum temperature for the chitinases was measured at temperatures ranging from 0 to 100 °C at pH 8.0 for *Zm*Chi19A and *Csp*Ch18A and pH 5.0 for *Os*Chi19A. To test for thermostability, protein samples were preincubated at temperatures ranging from 0 to 100 °C for 30 minutes, and the residual activity measured at 50 °C. The optimum pH was determined at 50 °C in buffers ranging from pH 2 to 13 in buffers A (25 mM sodium-citrate, pH 2 - 6), buffers B (25 mM Tris buffer, pH 7 and 8) and buffers C (25 mM Sodium-Carbonate, pH 9 – 13). To determine pH stability, the enzymes were preincubated at 0 °C for 30 minutes in buffers A-C with a pH range of 2.0–13.0 and the residual activity was measured at 50 °C in pH 8.0 (buffer B) for *Zm*Chi19A and *Csp*Ch18A and pH 5.0 (buffer A) for *Os*Chi19A (Horiuchi et al., 2016). For assays involving variations of pH and temperature, the 4-MU-(GlcNAc)_3_ was prepared in the identified buffer with the appropriate pH for the assay. The reactions were stopped by the addition of 100 µL of 1 M glycine/NaOH stop buffer to a final concentration of 1 mM glycine and fluorescence was measured immediately (Steentoft et al., 2013). The enzyme activity was calculated using an experimentally determined calibration curve to convert the change in fluorescence to the concentration of free 4-methylumbelliferone (4-MU) released. Fluorescence readings for all assays were measured at excitation 350 nm and emission 460 nm.

Colloidal chitin chitinolytic activities were assessed by incubating with 10 mg/mL colloidal chitin in 25 mM Tris-HCl buffer, pH 8.0 at 50 °C for 5 mins, supernatants were collected and mixed with 4 times volume of DNS, followed by heating at 95 °C. The resulting mixtures were further diluted 1:1 with distilled water, 100 uL of the final solution was added to a 96-well plate and the absorbance measured at 540 nm. All measurements were performed at room temperature in Falcon™ 96-well plates (Catalog# 351172 or 353948, Thermofisher Scientific, Waltham, MA) and read with a Synergy II plate reader (The Lab World Group, Hudson, MA) or Varian-Cary Eclipse Fluorescence Spectrophotometer (American Laboratory Trading, San Diego, CA) to obtain fluorescence spectra. All determinations were performed in triplicate and at least twice independently. For each incubation time, mixtures containing the same components (except for the protein) as the control condition with the SUMO protein, which provided the blank values (averages of triplicates) that were subtracted from the average value obtained in the presence of chitinases. The activity is expressed as µmol 4MU/min/mmol protein (Horiuchi et al., 2016).

### 2.11 Antifungal assay

A modified disk growth test was conducted to test the antifungal activity of wild-type and mutant chitinases. Fungal spores were resuspended in 25 mM Sodium Acetate, pH 5, imaged, and counted using ImageJ or Fiji software (National Institutes of Health, Bethesda, MD), and diluted to ~2.5 to ~5 spores/µL in 25 mM Sodium Acetate, pH 5 with the stated concentration of enzyme. The mixtures were incubated at 37°C for 30 min. Sample and spore mixtures were then applied to 5 mm sterile paper disks placed on potato dextrose agar in a 9 cm diameter Petri dish supplemented with 25 mg/mL chloramphenicol to inhibit bacterial growth. The plates were incubated at room temperature in a dedicated cell culture hood and photographed at 12 h intervals (Alastruey-Izquierdo et al., 2015; (Puškárová et al., 2017).

### 2.12 Statistical analysis

All experiments were performed in triplicates and error bars for standard deviation are shown in the figures. $Standard\;deviation=\;\sqrt{Se^{2}+Sb^{2}}$ where Se = standard deviation of the sample and Sb = standard deviation of the subtracted blank. The blank values were derived from buffer alone or SUMO in buffer measured in the same experiment and subtracted from all values before fitting. All binding isotherms were fit to the Langmuir equation $\left(\text{A}=\left({\text{A}}_{\text{max}}*\text{S}\right)/\left(\text{S}+K_{M}\right)\right)$ where A = the change in absorption at 450 nm per min with Amax being the maximum values and S = substrate concentration. Fittings and estimated of statistical significance were performed by Sigmaplot. All reported values for *K*_M_ passed the Normality (Shapiro-Wilk) and the Constant Variance (Spearman Rank Correlation) tests. One unit (U) of chitinase activity represents 1μmol of 4-MU released by enzyme from 4MU-GlcNAc_3_ per min under reaction conditions.

## 3 Results

### 3.1 Protein expression, assay conditions and substrate preferences

**Figure 1:**
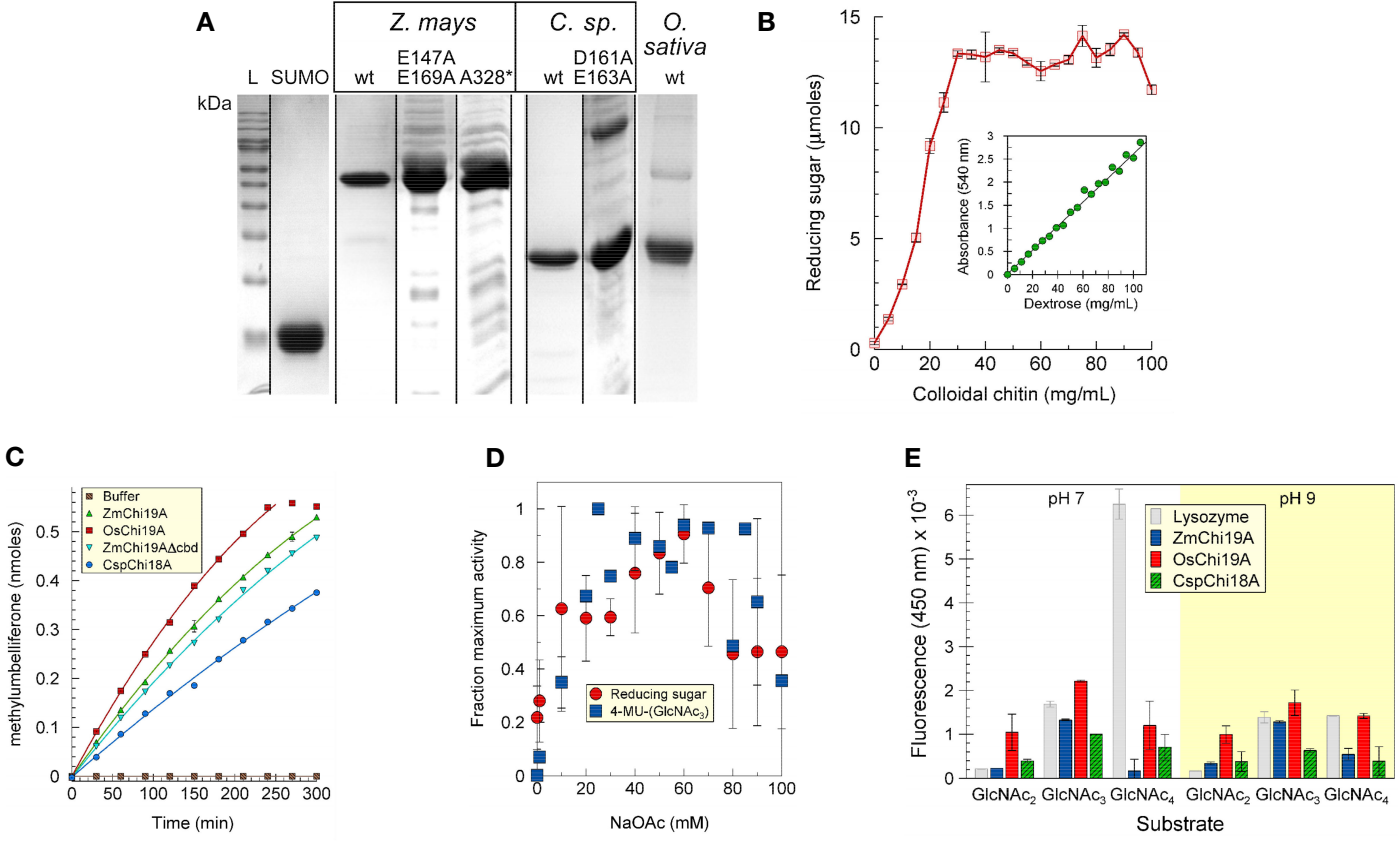
Purification and initial characterization of native chitinases and mutant forms. **(A)** SDS-PAGE analysis of purified SUMO, *Zm*Chi19A and mutants, *Csp*Chi18A and mutants, and *Os*Chi19A. Lane L, protein mass markers. **(B)** *Zm*Chi19A chitin cleavage activity as a function of substrate concentration (inset: standard curve). **(C)** Enzyme activity as a function of time. **(D)** Effect of salt concentration on enzyme activity. **(E)** Substrate preferences of lysozyme (gray), *Zm*Chi19A (blue), *Os*Chi19A (red) and *Csp*Chi18A (hatched green) using 4-MU-GlcNac_2_, 4-MU-GlcNac_3_, and 4-MU-GlcNAc_4_ as substrates. The graphs show the averaged data from three **(B, E)** and two **(D)** experiment performed in duplicate or triplicate (means ± SD). **(C)** shows the results from one experiment performed in triplicate for which there is one other experiment showing similar results.

The maize, rice chitinases and bacterial chitinases and some mutant versions were expressed in *E. coli*, purified, and analyzed by SDS-PAGE (**Figure 1A**). For most experiments a fusion protein of *Zm*Chi19A with an N-terminal SUMO solubility tag was used due to the limited solubility of untagged *Zm*Chi19A. Apparent molecular masses calculated from the Rfs after electrophoresis by SDS-PAGE were within 12% of those predicted from their amino acid sequences for all proteins (**Supplementary Table S3**).

The ability to cleave colloidal chitin was demonstrated for SUMO-*Zm*Chi19A (**Figures 1B**, **Supplementary Figure S1**) and *Csp*Ch18A (**Supplementary Figure S1**). A trimeric N-acetyl-glutamine substrate linked to methylumbelliferone (4-MU-GlcNAc_3_) was also a substrate for these chitinases (**Figure 1C**). The salt dependency of *Zm*Chi19A activity was similar when tested with chitin or 4-MU-GlcNAc_3_ as substrates (**Figure 1D**). To determine the optimal length of substrate, we compared the activities of *Zm*Chi19A and *Csp*Ch18A with 4-MU-GlcNAc_3_ substrates consisting of different multiples of N-acetyl-D-glucosamine. The GH19 chitinases, *Zm*Chi19A and *Os*Chi19A, prefer the 4-MU-GlcNAc_3_ at pH 7 and 9 (**Figure 1E**). Consequently, 4-MU-GlcNAc_3_ was used as the substrate for further enzymatic analysis.

### 3.2 Chitinase phylogenetic relationships and structures

**Figure 2:**
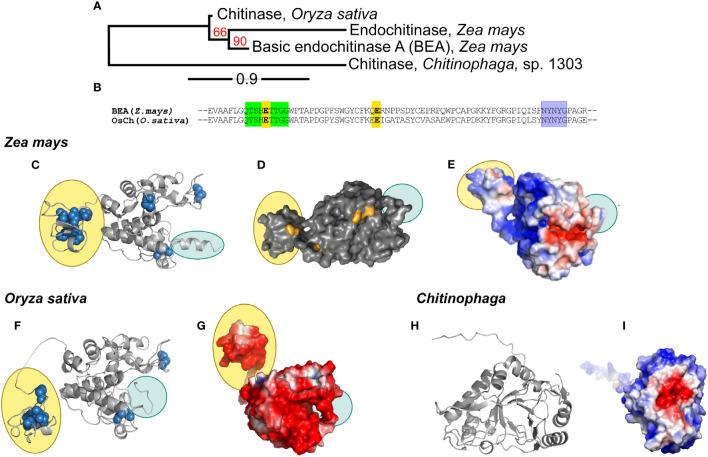
Phylogenetic analysis, conserved domains, structures, and surface charge distribution of rhizosphere associated chitinases. **(A)** Phylogenetic analysis of *Zm*Chi19A, *Os*Chi19A and *Csp*Chi18A. The red numbers on the branches are the probabilities of sharing a common ancestor determined by the bootstrap branch support. The line segment with the number ‘0.9’ shows the length of branch that represents the amount of evolutionary genetic change over time. **(B)** Sequence alignment of *Zm*Chi19A and *Os*Chi19A showing the conserved domains (in green and purple) and potential catalytic residues (in yellow). AlphaFold derived structures of **(C)** *Zm*Chi19A, secondary structure, **(D)** *Zm*Chi19A, surface structure showing potential glycosylation sites (in orange), **(E)** *Zm*Chi19A, electrostatic surface view, **(F)** *Os*Chi19A showing disulfide bridges (in blue spheres), **(G)** *Os*Chi19A, electrostatic surface view, **(H)** *Csp*Chi18A, secondary structure, **(I)** *Csp*Chi18A, electrostatic surface view. All electrostatic surface views show positively charged regions and negatively charged regions in blue and red respectively. Yellow-filled ovals identify the chitin binding domains (CBD) and blue-filled ovals identify the C-terminal flexible domains (CTD). **Supplementary Figure S2** compares the predicted glycosylation sites on the two plant chitinases.

**Figure 3:**
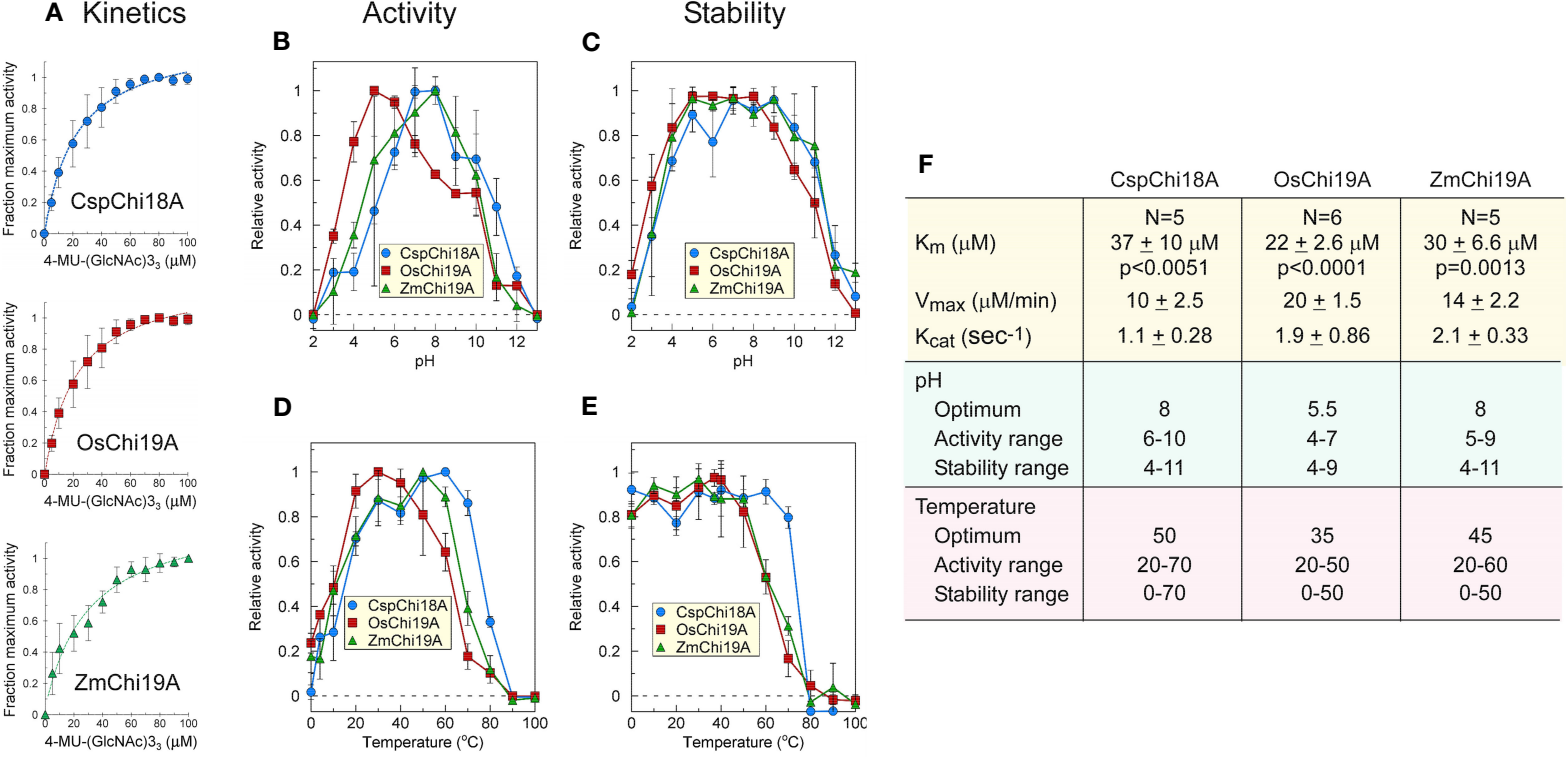
Catalytic activities of *Zm*Chi19A, *Os*Chi19A and *Csp*Chi18A using the soluble substrate (NAG)3-MUF. **(A)** Enzyme activity as a function of substrate concentration. Each curve shows the average of 5-6 independent experiments that were performed with 2 independently expressed and purified preparations of each enzyme **(B)** Effect of pH on chitinase activity. **(C)** Effect of pH on chitinase stability. **(D)** Effect of temperature on chitinase activity. **(E)** Effect of temperature on chitinase stability. **(F)** Table of derived kinetic constants. Data for B-E were from 2 independent experiments with enzymes from different expressed and purified preparations, each performed in triplicate. All values shown are the means ± SD.

A phylogenetic tree created from the multiple alignment of the maize, rice and bacterial chitinases (*Zm*Chi19A, *Os*Chi19A and *Csp*Ch18A respectively) predict that the maize and rice chitinases followed a similar evolutionary path but both lack orthology with the bacteria chitinase (**Figure 2A**). Tertiary structure predictions of the GH19 family chitinases, *Zm*Chi19A and *Os*Chi19A, identified the chitin-binding domain linked to an α-helix rich lysozyme-like catalytic domain with a deep cleft and a flexible C-terminal domain (**Figures 2C–G**). *Csp*Ch18A belongs to the GH18 family that is characterized by a catalytic region consisting of a triosephosphate isomerase (TIM) barrel (β/α) domain (**Figures 2H, I**). Despite both being GH19 chitinases, *Zm*Chi19A and *Os*Chi19A show contrasting surface charge distributions with the *Os*Chi19A surface dominated by negatively charged amino acid residues (**Figure 2G**) and *Zm*Chi19A having mostly positively charged surface residues on both the catalytic and chitin-binding domain (**Figure 2E**). All three chitinases have a predominantly negatively charged catalytic cleft (**Figures 2E, G, I**). The similar surface charge distributions of *Csp*Ch18A and *Zm*Chi19A differ greatly from that of *Os*Chi19A, which is highly negatively charged (**Figure 3G**).

### 3.3 Kinetic parameters, optimum pH, and temperature

Kinetic parameters (*K*_M_, V_max_, and K_cat_) were similar for all three chitinases (**Figures 3A, F**), although they were determined under different conditions (50°C, pH 8 for SUMO-*Zm*Chi19A and *Csp*Ch18A, and 40 °C, pH 5 for *Os*Chi19A). *Zm*Chi19A and *Csp*Ch18A showed a high pH optimum and retained at least 70% activity over a wide pH range (**Figures 3B, F**), whereas the pH optimum for *Os*Chi19A was lower with a smaller pH range over which it retained at least 70% of the maximum activity (**Figures 3B, F**). *Zm*Chi19A and *Csp*Ch18A were also stable (with at least 70% activity retained) to incubation for one hour over a larger pH range than *Os*Chi19A (**Figures 3C, F**). In addition to being more stable at higher temperatures (**Figures 3E, F**), *Zm*Chi19A and *Csp*Ch18A had higher temperature optima for catalysis than *Os*Chi19A (**Figures 3D, F**). All three enzymes were stable to cold temperatures (**Figure 3E**).

### 3.4 Key residues involved in catalysis

**Figure 4:**
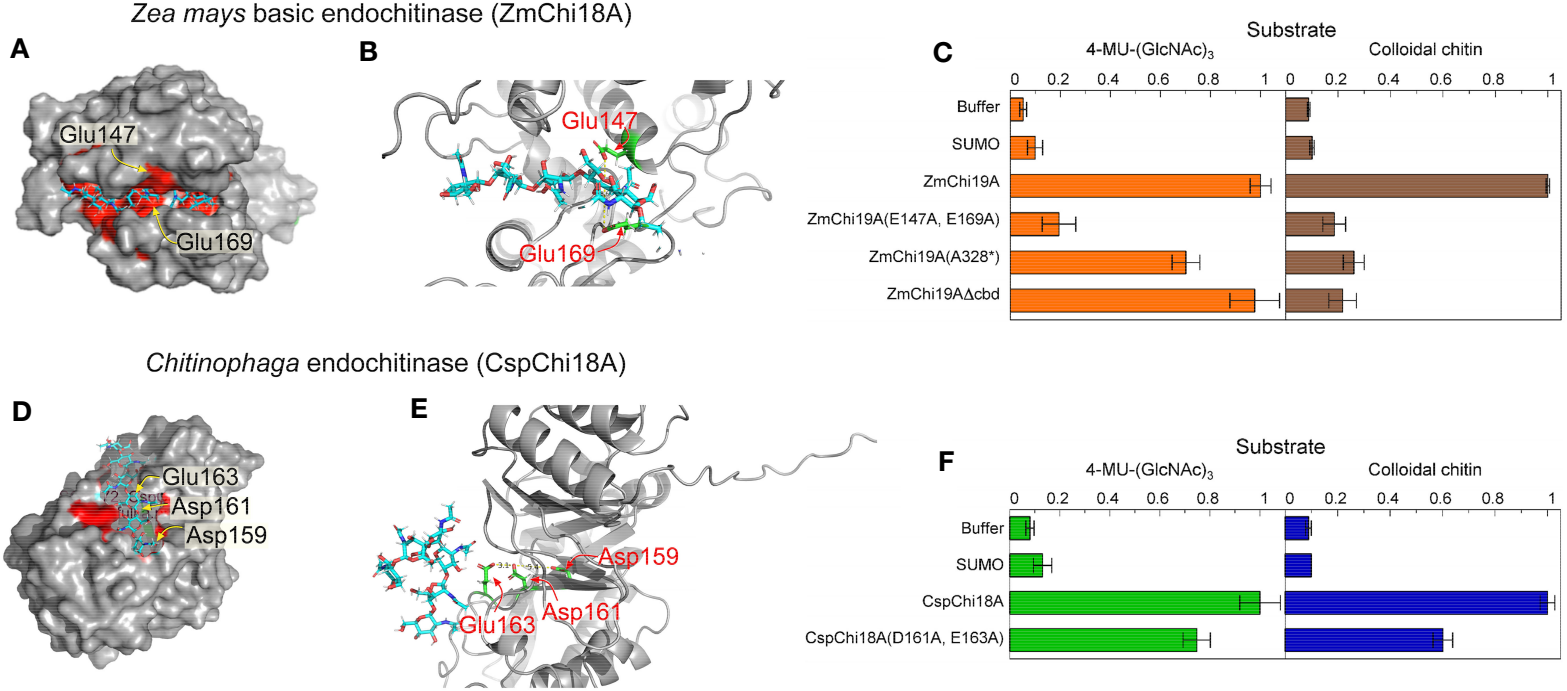
Amino acid residues in GH19 and GH18 chitinases for substrate binding and catalysis. The substrate (GlcNAc)_6_ is shown as cyan sticks in all models. Model structures were created with alphFold **(A)** Surface structure of *Zm*Chi19A with the catalytic site in red and predicted catalytic residues labeled, **(B)** Structure of the *Zm*Chi19A catalytic site with predicted catalytic residues in green. **(C)** Activities of the wild-type *Zm*Chi19A and its mutants against substrates 4-MU-(GlcNAc)_3_ and colloidal chitin. **(D)** Surface structure of the *Csp*Chi18A catalytic site in red with predicted catalytic residues labeled. **(E)** Structure of the *Csp*Chi18A catalytic site with predicted catalytic residues in green. **(F)** Activities of the wild-type CspCh and its mutant against substrates 4-MU-(GlcNAc)_3_ and colloidal chitin. The graphs show the average results from 6 (4-MU-(GlcNAc)_3_) and 2 (chitin) experiments, each performed independently in triplicate. The background of fluorescence with SUMO was not subtracted from all values to show the value of the SUMO control compared with buffer and other activities. Whereas the activity of *Zm*Chi19A and *Csp*Chi18A are shown relative to the native activities, the rate of chitin cleavage by *Csp*Chi18A is 27% of the rate of cleavage by BEA when both are normalized to the baseline rate of SUMO.

To investigate the residues essential for catalysis of the GH19 and GH18 chitinases, we modeled the structure of *Zm*Chi19A and *Csp*Ch18A in complex with the substrate (chitin, (GlcNAc)_6_) by molecular docking. In the modeled structures, (GlcNAc)_6_ is bound in the negatively charged substrate cleft with the average distance of substrate to the predicted catalytic residues being 3.1 Å (**Figures 4A, D**). In *Zm*Chi19A, the scissile glycosidic bond is sandwiched between the side chain of the conserved and predicted catalytic residues Glu147 and Glu169, which are 8.6 Å apart (**Figures 2B**, **4B**). In *Csp*Ch18A, Asp161 and Glu163 were identified as potential catalytic residues (**Figures 4D, E**). To test the predicted catalytic roles, *Zm*Chi19A and *Csp*Ch18A were produced with alanine substitutions for these residues and the activities of these mutant proteins were compared with the wild-type enzymes using both 4-MU-GlcNAc_3_ and colloidal chitin (**Figures 4C, F**). The mutants of *Zm*Chi19A and *Csp*Ch18A were also folded with AlphaFold and aligned with the native enzyme to evaluate the effect of the mutations on enzyme structures. These comparisons showed that the folded structures of all mutants were within 1.5 RMSD of the relevant native structure (**Supplementary Figure S3**). *Zm*Chi19A(E147A,E169A) showed almost complete loss of activity, which supports a key role of these residues in catalysis. Loss of the chitin binding domain or the C-terminal domain had little effect on the ability of *Zm*Chi19A to cleave the soluble substrate but effectively eliminated the ability to cleave colloidal chitin (**Figure 4C**). By contrast, *Csp*Ch18A(D161A,E163A) retained ~60% of the activity of the native enzyme in enzymatic assays using either 4-MU-GlcNAc_3_ and colloidal chitin, which suggests a potential ancillary role in catalysis (**Figure 4F**).

### 3.5 Plant root chitinase impact on fungal growth

**Figure 5:**
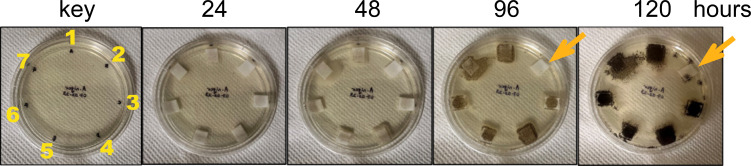
Antifungal activity assay of purified recombinant native and mutant chitinases towards *Aspergillus niger*. Starting as spores (~5/filter), *Aspergillus niger* was incubated with 50 ug (0.5 mg/mL) of enzyme at room temperature (23-24 °C) over a period of 5 days. 1) SUMO, 2) SUMO-*Zm*Chi19A, 3) SUMO-*Zm*Chi19A(E147A,E169A), 4) SUMO-ZmChi19A(A328*), 5) *Zm*Chi19AΔCBD, 6) *Csp*Chi18A, and 7) *Csp*Chi18A(D161A,E163A). This data is representative of the results obtained in six independently performed experiments.

The three chitinases and their mutated and truncated versions were tested for their activity against the fungus *Aspergillus niger* (**Figure 5**). Only the native (full-length) *Zm*Chi19A inhibited the fungal growth. This result and their requirement for colloidal chitin cleavage shows that, although the CBD and flexible C-terminal domain are not necessary for catalysis, they are essential for anti-fungal activity, which requires chitin cleavage.

## 4 Discussion

### 4.1 Predicted structure and surface charge distribution

Based on sequence similarity, plant chitinases are classified into seven classes (I–VII). Classes represented in the GH19 family are I, II, IV, VI and VII, while classes III and V are members of the GH18 family. *Zm*Chi19A and *Os*Chi19A are maize and rice root endochitinases respectively, and members of the GH19 family to which most plant chitinases belong (Oyeleye and Normi, 2018; Zhou et al., 2023), while *Csp*Ch18A is a symbiotic bacterial endochitinase in the GH18 family (Oyeleye and Normi, 2018; Haxim et al., 2022). GH18 and GH19 chitinases commonly use different catalytic mechanisms, producing different configuration of products (Funkhouser and Aronson, 2007; Oliveira et al., 2020). All GH18 chitinases are characterized by a catalytic region that consists of a triosephosphate isomerase (TIM) barrel (β/α)8 domain, while the catalytic domain of family GH19 is an α-helix rich lysozyme-like domain characterized by a deep cleft (Wang et al., 2019). Class I endochitinases are characterized by the presence of an N-terminal hevein-like chitin-binding domain (CBD) (Horiuchi et al., 2016) and a C-terminal catalytic domain (CatD), which are connected by a short linker that varies in length and amino acid composition. The *Zm*Chi19A and *Os*Chi19A chitinases have a CBD and thus are classified as type I.

Despite having similar structures and conserved domains *Zm*Chi19A and *Os*Chi19A have contrasting surface charge distributions and different pH optima, which might be adaptations to different environments. Maize plants are C4 plants (Bellasio et al., 2023) more suited to hot climates while rice plants are C3 plants which grow in cool environments (Bellasio et al., 2019; Osinde et al., 2023). *Oryza sativa* normally grows emerged in water (Yan et al., 2023), whereas *Zea mays* grows under more dry conditions on land. Differences in the charge distributions of *Zm*Chi19A and *Os*Chi19A may be adaptations to specific ecological niches, lifestyles, or host-pathogen interactions (Vanhoye et al., 2004; Garcia-Moreno, 2009; Gerland et al., 2020; Kim et al., 2020; Panja et al., 2020; Di Savino et al., 2021; Doan et al., 2022; Vallina Estrada et al., 2023).

### 4.2 Enzyme activity and stability

*Zm*Chi19A and *Csp*Ch18A degraded colloidal chitin (*Os*Chi19A was not tested) and all three chitinases degraded soluble chitin substrates, which suggests that they may play an important role in chitin recycling and defense against harmful fungi. The recombinant enzymes were tested for substrate preference and their kinetic parameters were determined using the preferred substrate, 4-MU-(GlcNAc)_3_. The obtained data were in the range of those reported for several chitinases as summarized in **Supplementary Table S4**. For example, the *K*_M_ values for *Zm*Chi19A, *Os*Chi19A and *Csp*Ch18A were 30, 22 and 37 uM respectively, which are similar to 33 uM for a barley chitinase (Kuo et al., 2008), 42 uM for a *S. marcescens* chitinase (Honda et al., 2003), and 49 uM for an *Aspergillus niger* chitinase (van Munster et al., 2015). Higher *K*_M_ values were reported for chitinases from rubber (*Hevea brasiliensis*) and toxic plant (weed) *Ipomoea carnea* (Patel et al., 2009; Sukprasirt and Wititsuwannakul, 2014, **Supplementary Table S4**).

The recombinant enzymes were also characterized for pH and temperature optima and stabilities. The optimum pH and temperature ranges over which these chitinases were stable were in the range of those reported for other reported plant, fungi and bacterial chitinases (Patel et al., 2009; Sukprasirt and Wititsuwannakul, 2014; Horiuchi et al., 2016; Thimoteo et al., 2017; Wang et al., 2019; Rajninec et al., 2020, **Supplementary Table S4**). The pH optimum and pH range for *Os*Chi19A activity are consistent with those reported for other plant chitinases (Patel et al., 2009; Sukprasirt and Wititsuwannakul, 2014; Horiuchi et al., 2016; Sierra-Gómez et al., 2019; Wang et al., 2019). However, unlike other reported plants and bacterial chitinases, all of which have reported pH optima between 4.5 and 6, *Zm*Chi19A and *Csp*Ch18A were most active at pH 8.

### 4.3 Catalytic residues and domains

The conserved catalytic residues of GH19 chitinases Glu147 and Glu169 were confirmed to play a key role in catalysis by *Zm*Chi19A due to their mutagenesis resulting in the complete loss of enzymatic activity against colloidal chitin, the soluble trimeric saccharide and *Aspergillus niger*. The identification of catalytic residues in *Csp*Ch18A based on the conservation of sequence with other GH18 chitinases combined with molecular docking of the substrate to the alphaFold modeled *Csp*Ch18A suggested that Asp161 and Glu163 were potential catalytic residues (Wang et al., 2019). However, with both residues mutated, the enzyme retained ~60% of its activity. Thus, these residues are not critical for catalysis.

Modeling by alphaFold identified the flexible C-terminal domain (CTD), which proved to be essential for *Zm*Chi19A to cleave colloidal chitin and inhibit fungal growth. This is the first report showing the importance of the CTD in the role of plant chitinases. Only the CBD has been previously shown to be required for activity (Onaga and Taira, 2008; Huang et al., 2009; Yokoyama et al., 2009). We propose that the CTD might provide a second site of interaction with chitin in addition to the CBD or hold chitin in the catalytic cleft for enzymatic degradation, thereby enhancing the efficiency of chitin breakdown in the soil. In another example, a mobile cap domain identified in a family IV esterase from sorghum rhizosphere microbiome was proposed to regulate substrate access (Distaso et al., 2023).

### 4.4 Roles of plant root and associated chitinases in the rhizosphere

The ability of *Zea mays Zm*Chi19A to inhibit the growth of the pathogenic fungus, *Aspergillus niger*, is consistent with the hypothesis that GH19 chitinases like *Zm*Chi19A become part of the plants’ defense arsenal against harmful soil fungi when released by plant roots (Huang et al., 2009; Yokoyama et al., 2009; Haldar and Sengupta, 2015). By contrast, the lack of ability of the plant root associated GH18 bacteria chitinase to inhibit *Aspergillus niger* growth is consistent with the hypothesis that it is responsible for downstream chitin degradation rather than being part of an initial defense again fungal pathogens in the rhizosphere (Huang et al., 2009; Roy et al., 2021; Chandra et al., 2022; Kotb et al., 2023). However, as we have only investigated the effects of these chitinases on one fungus in this study, it may also be that the bacterial chitinase is effective against some but not all fungi. Further investigation is required to fully understand the potential roles of these chitinases in plant defense.

In addition to their involvement in plant defense, plant root chitinases may play a role in plant growth and development by digesting chitin in the rhizosphere and releasing nutrients (Chandra et al., 2022). Chitinase activity in the rhizosphere can release nitrogen from chitin-containing residues, such as dead insects or fungal hyphae, providing more nitrogen to plants and potentially reduce the need for synthetic fertilizers.

Further understanding the mechanisms underlying plant-microbe interactions and roles of root chitinases in rhizosphere ecology can have many positive impacts on agriculture including to 1) support the development of more ecologically friendly and sustainable farming techniques, 2) decrease reliance on chemical pesticides and fertilizers, 3) improve nutrient cycling, and 4) support the development of transgenic crops with enhanced chitinase activity as an alternative to chemical fungicides (Oyeleye and Normi, 2018; Wang et al., 2019). This knowledge will also contribute to biotechnology applications, including the design of more effective chitin-degrading enzymes for the industrial processing of chitin (Krolicka et al., 2018; Karnaouri et al., 2019). They can also be developed as *in situ* biomarkers (Meirinho et al., 2016) in studies to understand root behavior during plant stress and disease so as to improve overall plant health and crop productivity (Yim et al., 2022).

## Data availability statement

The raw data supporting the conclusions of this article will be made available by the authors, without undue reservation.

## Author contributions

SS: Conceptualization, Data curation, Formal analysis, Investigation, Methodology, Software, Supervision, Validation, Visualization, Writing – original draft, Writing – review & editing. OZ: Conceptualization, Investigation, Supervision, Writing – review & editing. MN-H: Data curation, Formal analysis, Funding acquisition, Investigation, Methodology, Project administration, Resources, Supervision, Validation, Visualization, Writing – review & editing.
