# Supplementary material for: Plant root associated chitinases: structures and functions
Source: Front Plant Sci. 2024 Feb 1;15:1344142. doi: 10.3389/fpls.2024.1344142 (PMC10867124; doi:10.3389/fpls.2024.1344142)
Supplement: Supplementary file 1 [file DataSheet_1.pdf]

Gel # 22908

Coomassie-blue stain

15% resolving SDS-PAGE, 5% stacker Tris-HCl Bis-acrylamide gel, ~10 µg proteins

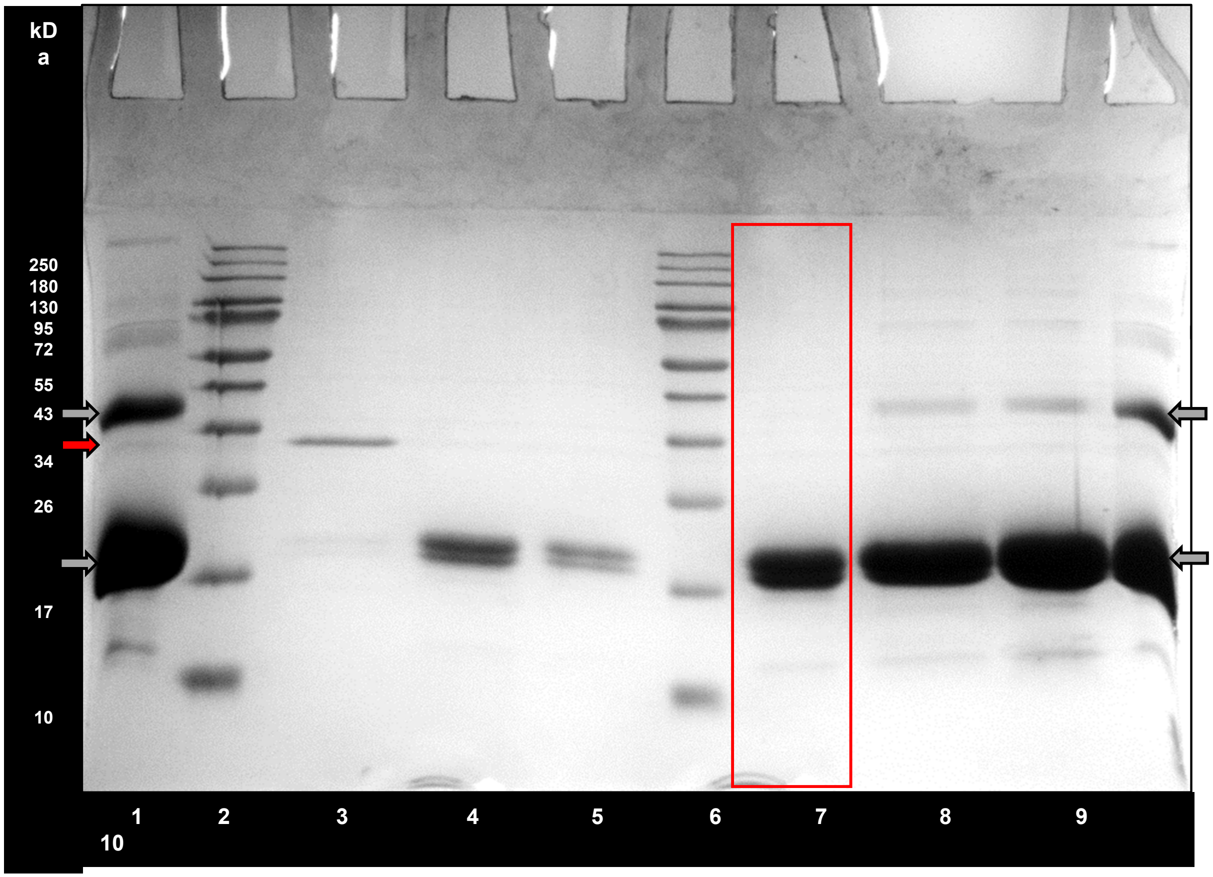

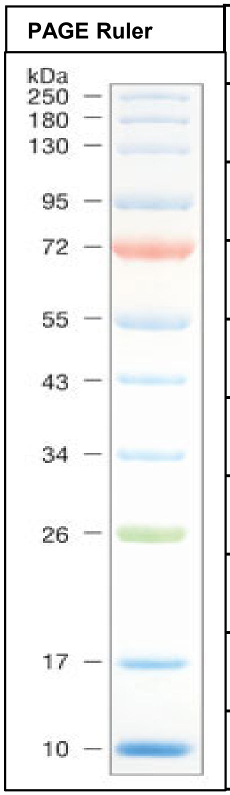

1	p28b-SUMO unpurified
2	Protein Ladder
3	p28a-CspCh [FPLC]
4	p28b-SUMO [FPLC]
5	p28b-SUMO [FPLC]-diluted
6	Protein Ladder
7	p28b-SUMO [FPLC+dialysis] conc.
8	p28b-SUMO [Dialysed]
9	p28b-SUMO [Dialysed conc.]
10	p28b-SUMO unpurified

1. p28b-SUMO: MW = 16.84 kDa, Dimer = 33.68 kDa
2. **p28a-CspCh:** **MW = 33.14 kDa, Dimer = 66.28 kDa**

SDS-PAGE gel of purified SUMO solubility tag

11/24/2023

Gel # 22906

Coomassie-blue stain

15% resolving SDS-PAGE, 5% stacker Tris-HCl Bis-acrylamide gel, ~10 µg proteins

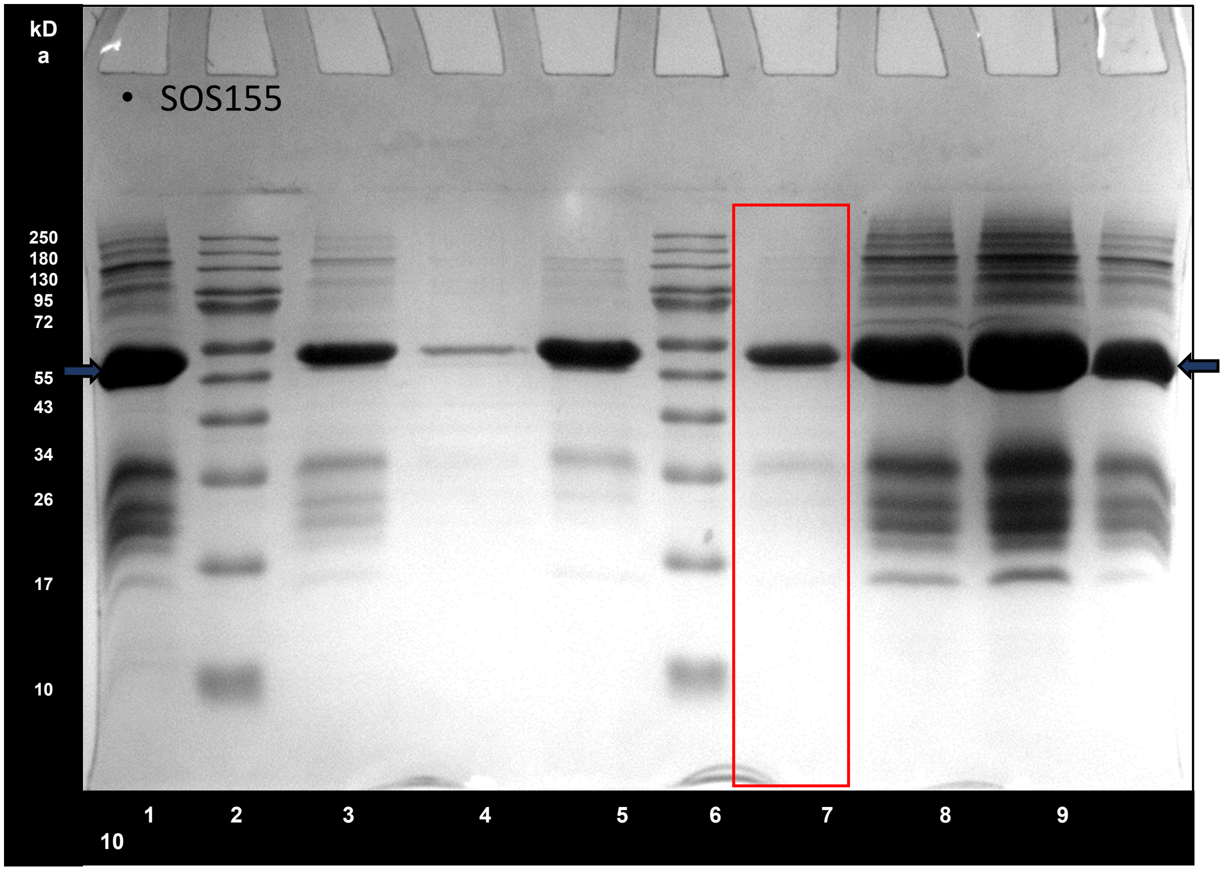

PAGE Ruler

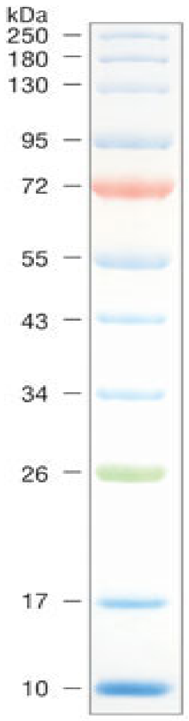

1	p28b-S-BEA unpurified
2	Protein Ladder
3	p28b-S-BEA unpurified
4	p28b-S-BEA [FPLC]
5	p28b-S-BEA [FPLC]-conc
6	Protein Ladder
7	p28b-S-BEA [FPLC+Dialysis]-conc
8	p28b-S-BEA [Dialysis]
9	p28a-CspCh [Dialysis]-conc
10	p28b-S-BEA unpurified

1. p28b-SUMO-BEA: MW = 47.53 kDa, Dimer = 95.06 kDa

SDS-PAGE gel of purified BEA

11/24/2023

Gel # 22986

Coomassie-blue stain

12 % resolving SDS-PAGE, 5% stacker Tris-HCl Bis-acrylamide gel, ~10 µg proteins

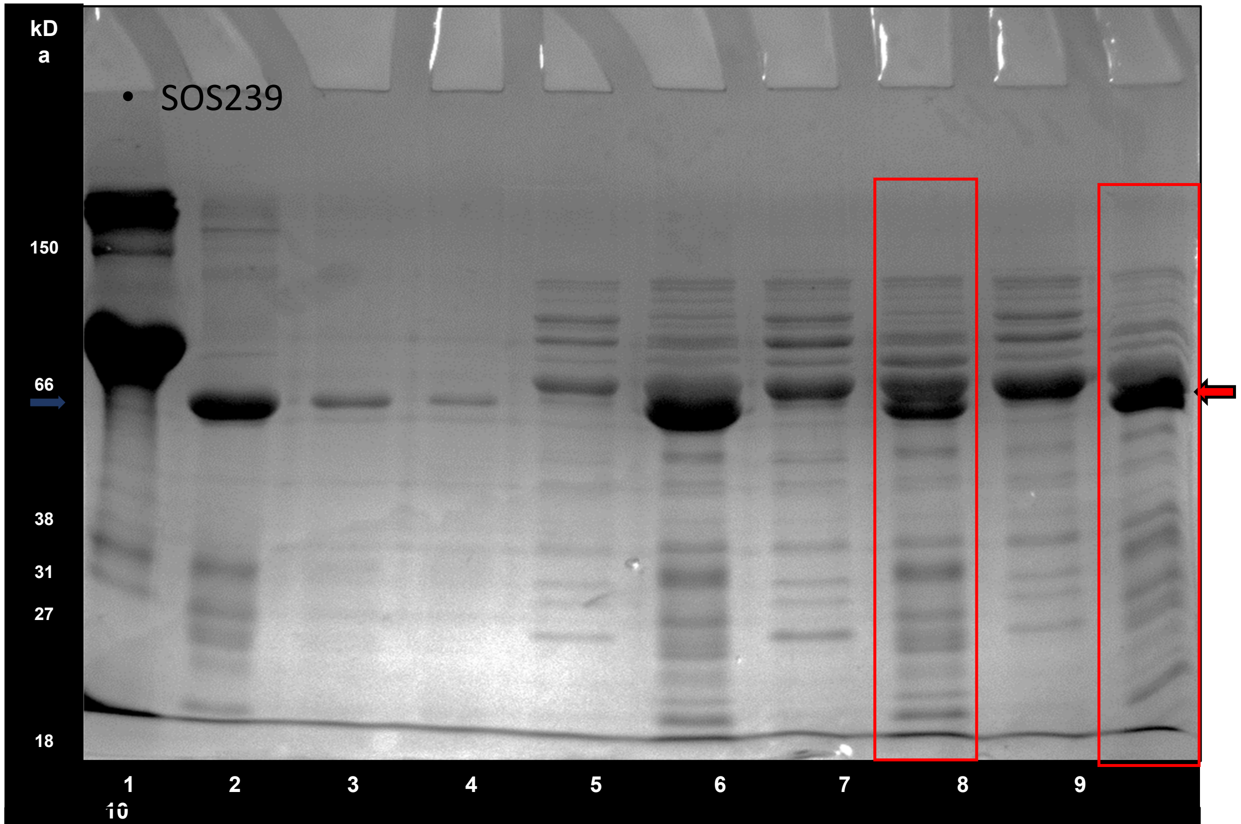

1. His-SUMO-BEA: MW = 47.53 kDa
2. His-SUMO-BEA_E147A, E169A: MW = 47.41 kDa
3. His-SUMO-BEA_A328*: MW = 46.86 kDa

PAGE Ruler

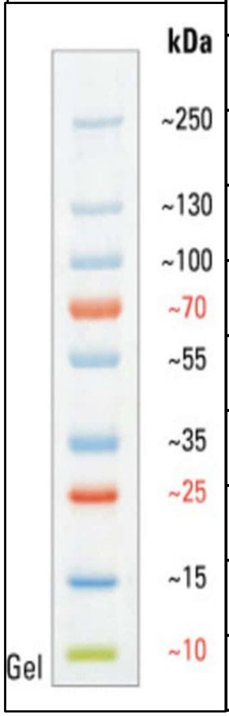

1	Protein mix ladder
2	His-SUMO-BEA FPLC purified (SOS 160)
3	His-SUMO-BEA FPLC purified (SOS 160) (4x diluted)
4	His-SUMO-BEA_A328* (dialysed & 4x diluted)
5	His-SUMO-BEA (non-dialysed)
6	His-SUMO-BEA (dialysed & conc.)
7	His-SUMO-BEA_E147A, E169A (non-dialysed)
8	His-SUMO-BEA_E147A, E169A (dialysed & conc.)
9	His-SUMO-BEA_A328* (non-dialysed)
10	His-SUMO-BEA_A328* (dialysed & conc.)

Dialysis of maize BEA & its mutants

11/24/2023

Gel # 22915

Coomassie-blue stain

12 % resolving SDS-PAGE, 5 % stacker Tris-HCl Bis-acrylamide gel, ~10 µg proteins

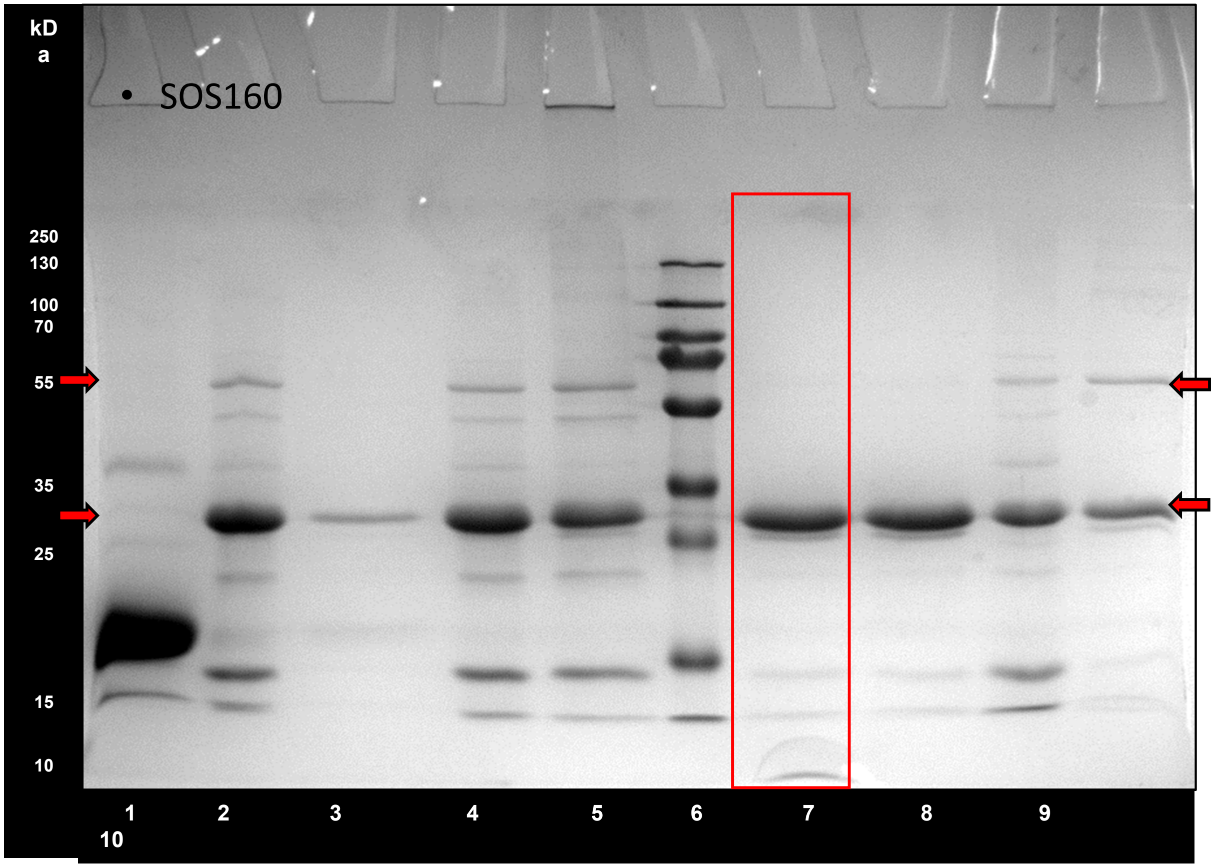

PAGE Ruler

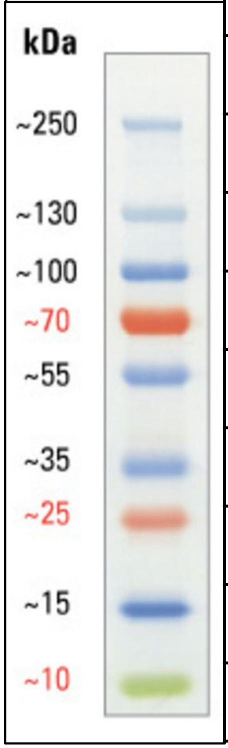

1	p28b-SUMO
2	p28a-CspCh [Dialysis]
3	p28a-CspCh [FPLC]-conc (SOS151)
4	p28a-CspCh [Dialysis]-conc
5	p28a-CspCh unpurified
6	Protein Ladder
7	p28a-CspCh B-4
8	p28a-CspCh [FPLC+Dialysis]-conc
9	p28a-CspCh [Dialysis]
10	p28a-CspCh [FPLC+Dialysis]-diluted

1. **p28a-CspCh: MW = 33.14 kDa, Dimer = 66.28 kDa**
2. **p28b-SUMO: MW = 16.84 kDa, Dimer = 33.68 kDa**

SDS-PAGE gel of purified CspCh

11/24/2023

Gel # 22987

Coomassie-blue stain

12 % resolving SDS-PAGE, 5% stacker Tris-HCl Bis-acrylamide gel, ~10 µg proteins

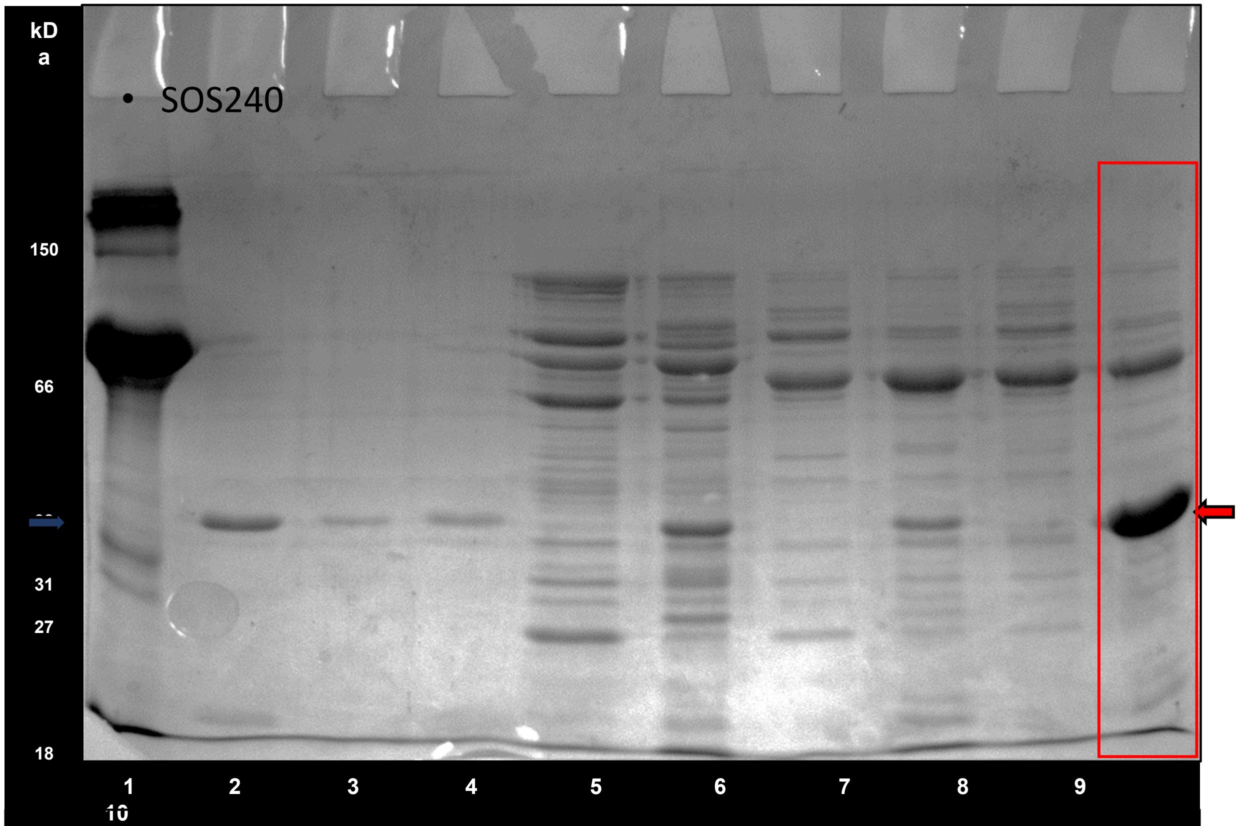

1. His-CspCh: MW = 33.14 kDa
2. His-CspCh_D161A, E163A: MW = 33.04 kDa

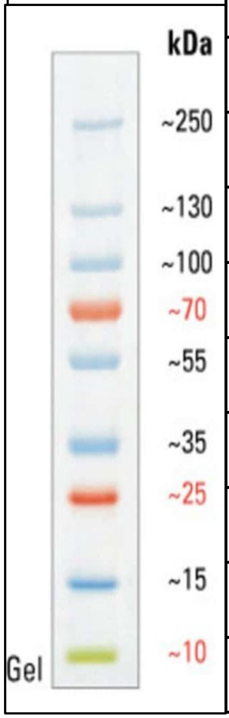

1	**Protein mix ladder**
2	His-CspCh FPLC purified (SOS 155)
3	His-CspCh FPLC purified (SOS 155) (4x diluted)
4	His-CspCh_D161A, E163A (dialysed & 4x diluted)
5	His-CspCh-OE (non-dialysed)
6	His-CspCh-OE (dialysed & conc.)
7	His-CspCh-RG2 (non-dialysed)
8	His-CspCh-RG2 (dialysed & conc.)
9	His-CspCh_D161A, E163A (non-dialysed)
10	His-CspCh_D161A, E163A (dialysed & conc.)

Dialysis of bacteria chitinase & its mutant

11/24/2023

Gel # 22841

- SOS114, 121, 155, 160

12% resolving SDS-PAGE, 5% stacker Tris-HCl Bis-acrylamide gel, ~15 µg proteins

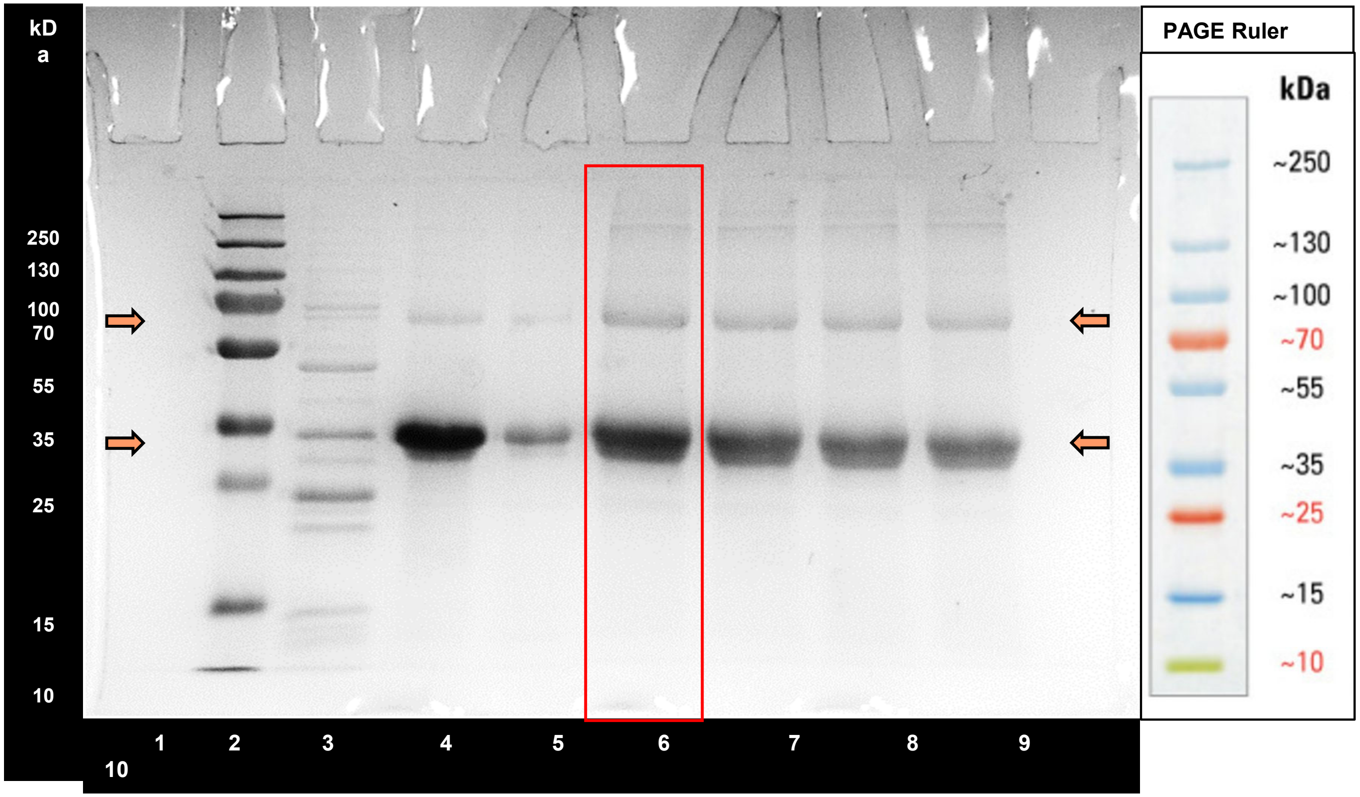

1	Refolding Buffer
2	Protein Ladder
3	OsCh [E1] -Cyt
4	OsCh [E1] -IBs
5	OsCh [E3] -IBs
6	OsCh [Refolded] –in RB
7	OsCh [Refolded] –in RB
8	OsCh [Refolded] –in BC100
9	OsCh [Refolded] –in BC100
10	Elution Buffer

1. p28a-OsCh: MW = 33.72 kDa, Dimer = 67.44 kDa

Refolding of OsCh from IBs of OverExpress C43(DE3) Cells

11/24/2023
