# Supplementary material for: Plant root associated chitinases: structures and functions
Source: Front Plant Sci. 2024 Feb 1;15:1344142. doi: 10.3389/fpls.2024.1344142 (PMC10867124; doi:10.3389/fpls.2024.1344142)
Supplement: Supplementary file 2 [file DataSheet_2.pdf]

Supplementary Material

Plant Root Associated Chitinases: Structures and Functions

Samuel O. Shobade[1,2], Olga A. Zabotina[1,2], Marit Nilsen-Hamilton[1,2*]

[1] Ames National Laboratory, U. S. Department of Energy, Ames, Iowa, USA,

[2] Roy J. Carver Department of Biochemistry, Biophysics and Molecular Biology, Iowa State University, Ames, Iowa, USA

*** Correspondence:** Marit Nilsen-Hamilton: marit@iastate.edu

1 Supplementary Tables

Table S1

S/N	Oligo	Sequence (5' – 3')
Cloning of *Zm*CHi19A into p28b-SUMO vector:		
1	T-T-BasicEndoA F	ATTGGTGGATCCGAGAACCTGTACTTTCAGGGCCAGCAGTGCGGGCAGCAG
2	T-BasicEndoA R	GGCCGCAAGCTTTCTATCATTACTGCGCCGCCACCTCAACCGCCAGT
Cloning of *Os*Chi19A into p28a vector:		
3	Oligo 6467 (O/CsChit-F)	CAATTCCCCTCTAGAAATAATTTTGTTTAACTTTAAGAAGGAGATATACC
4	Oligo 6468 (OsChit-R)	GTGGTGCTCGAGTTACTGCACAGCAG
Cloning of *Csp*Chi18A into p28a vector:		
5	Oligo 6467 (O/CsChit-F)	CAATTCCCCTCTAGAAATAATTTTGTTTAACTTTAAGAAGGAGATATACC
6	Oligo 6469 (CsChit-R)	GTGGTGCTCGAGTTAGGGCGTATACG
Cloning of *Zm*Chi19AΔcbd into p28a vector:		
7	Oligo 6472 (p28pp-BEAnCBD-F1)	CATGAGAGGATCGCATCACCATCACCATCACGGATCCATGAGAGACCTCTTCGAGCGGC
8	T-BasicEndoA R	GGCCGCAAGCTTTCTATCATTACTGCGCCGCCACCTCAACCGCCAGT
Primer for M4 deletion of C-terminal domain of *Zm*Chi19A:		
9	Oligo 6514 (S-BEA_A328*-R)	GGCCGCAAGCTTTCTATCATTACTGCGCCGCCACCTCAACTCACAGTCCGCTATTGAAGG
Site-Directed Mutagenesis: of *Csp*Chi18A:		
10	Forward Primer Oligo 6510 (D161A_E163A-F)	CTGGATGGCATTGATTTCGCGGACGCGTATGCCGACTATGGTAAC
11	Reverse Primer Oligo 6511 (D161A_E163A-R)	GTTACCATAGTCGGCATACGCGTCCGCGAAATCAATGCCATCCAG
p28 Vector Colony PCR & Sequencing Primers		
12	Oligo 6470 PETFOR (for pET vector)	TAATACGACTCACTATAGGG
13	Oligo 6471 PETREV (T7 Terminator for pET vector)	GCTAGTTATTGCTCAGCGG

Legend: Sequences of oligonucleotides used in this work.

Table S2

Enzyme	Prep#	FPLC-purified	% purity
SUMO	SOS155	Yes	99%
	SOS160	No	93%
SUMO-*Zm*Chi19A	SOS155	Yes	97%
	SOS239	No	77%
*Csp*Chi18A	SOS160	Yes	100%
	SOS 240	No	76%
*Os*Chi19A	SOS 114	No	95%
	SOS 129	No	97%
	SOS 124	No	73%
SUMO-*Zm*Chi19A(E147A, E169A)	SOS 239	No	84%
	SOS 307	No	76%
SUMO-*Zm*Chi19A(A328*)	SOS 239	No	94%
	SOS 307	No	81%
*Csp*Chi18A(D161A, E163A)	SOS 240	No	91%
	SOS 307	No	92%

Legend: Estimated purities of preparations used in this work. Samples of each preparation were resolved by SDS PAGE through 12% or 15% acrylamide gels depending on the molecular size of the protein (see examples in Figure 2). The relative area under each protein peak was determined by ImageJ analysis of the Coomassie blue stained gels. The % purity was determined from percent of the total quantified protein that was found in the band representing the purified protein. It should be noted that, because the gels were loaded with sufficiently high amounts of protein to detect contaminant proteins and the contaminants are at such lower levels than the purified protein, the Coomassie blue signal is likely not linear with amount of protein over the entire range of measurement. Thus, these estimates of purity are likely to be lower than the actual values and represent minimum % purity for each preparation.

Table S3

Sample	Obtained Average	Obtained S.D	Obtained c.v.	Expected	Obtained/calculated
SUMO	19	1.0	5%	16.8	1.1
SUMO-*Zm*Chi19A	42	1.0	2%	47.5	0.88
*Os*Chi19A	32	1.22	4%	33.7	0.93
*Csp*Chi18A	31	3.94	7%	33.1	0.94

Legend: Estimated molecular weights compared with those determined from the cloned protein sequences. The obtained values for molecular weight (kDa) were determined for each of the listed proteins by from their relative mobilities (Rfs) on SDS PAGE using a set of standard proteins run on the same gels. The log MW of the standards was plotted against their Rfs and the curves fit with a polynomial (order 3). Values were determined from 3 separately run gels for each protein to provide the average estimates of the MW with standard deviation (S.D.) and coefficient of variation (c.v.).

Table S4

Enzyme, Substrate	K_m (µM)	V_{max} (µMmin^{-1})	K_{cat} (Sec^{-1})	pH			Temperature (^{0}C)			Source
				Optima	Activity Range	Stability Range	Optima	Activity Range	Stability Range	
**Plant chitinases**										
*Zea mays: Zm*Chi19A 4-MU-(GlcNAc)$_3$	30 ± 6.6	14 ± 2.2	2.1 ± 0.33	8.0	5.0 – 9.0	4.0 –11.0 (1 h)	45	20 - 60	0 - 50 (1 h)	This work
*Oryza sativa: Os*Chi19A 4-MU-(GlcNAc)$_3$	22 ± 2.6	20 ± 1.5	1.9 ± 0.86	5.5	4.0 – 7.0	4.0 – 11.0 (1 h)	35	20 - 50	0 - 50 (1 h)	This work
Barley chitinase, 4-MU-(GlcNAc)$_3$	33	12	0.006	5.0	4.0 –7.0	-	-	-	-	(Hollis et al., 1997)
Barley chitinase, 4-MU-(GlcNAc)$_4$	3	1.2	0.58	5.0	4.0 –7.0	-	-	-	-	(Hollis et al., 1997)
Rubber *(Hevea brasiliensis)* chitinase, 4-MU-(GlcNAc)$_3$	100	-	-	5.0	4.0 – 7.0	4.0 – 7.0 (20 min)	45	30 - 40	30 - 40 (20 min)	(Sukprasirt and Wititsuwannakul, 2014)
Ipomoea carnea chitinase, p-np-(GlcNAc)	500	0.025	29	6.0	5.0 – 9.0	5.0 – 9.0 (24 h)	60	60 - 80	20 - 80 (1 h)	(Patel et al., 2009)
Drosera rotundifolia chitinase, FITC-Chitin	1660	88.50	77	6.0	5.0 – 6.0	-	40	20 - 40	-	(Rajninec et al., 2020)
Agave tequilana chitinase, CC	-	-	-	5.0	2.0 – 7.0	2.0 – 9.0 (24 h)	-	-	-	(Sierra-Gómez et al., 2019)
**Fungal chitinase**										
Aspergillus niger chitinase, 4-MU-(GlcNAc)$_3$	50 ± 6.9	-	16± 0.9	-	-	-	-	-	-	(van Munster et al., 2015)
**Bacterial chitinases**										
Chitinophaga oryzae 1303, *Csp*Ch18A 4-MU-(GlcNAc)$_3$	37 ± 10	10 ± 2.5	1.1 ± 0.28	8.0	6.0 –10.0	4.0 –11.0 (1 h)	50	20 - 70	0 - 70 (1 h)	This work
Pseudoalteromonas aurantia Chi23, 4-MU-(GlcNAc)$_2$	65± 12	0.011	5.4 ± 0.5	5.0	4.0 –5.0	3.0 –10.0 (1 h)	60	50 - 70	50 - 70 (1 h)	(Wang et al., 2019)
S. marcescens ChiA, 4-MU-(GlcNAc)$_2$	59	-	35	-	-	-	-	-	-	(Honda et al., 2003)
S. marcescens ChiB, 4-MU-(GlcNAc)$_2$	42	-	12	-	-	-	-	-	-	(Honda et al., 2003)
Thermococcus chitonophagus, Tc-ChiD(ΔS), CC	-	-	-	4.0	3.5 – 4.5	-	90	90 - 95	80 - 100 (30 min)	(Horiuchi et al., 2016)
MetaChi18A chitinase, 4-MU-(GlcNAc)$_2$	2.7	40	3.2	5.0	4.0 – 9.0	4.0 –11.0 (1,24 h)	50	40 - 60	20 - 50 (1,3,24 h)	(Thimoteo et al., 2017)
Thermococcus chitonophagus, Tc-ChiD(ΔBD): CC	-	-	-	4.5	4.5 – 5.0	-	80	80 - 85	80 - 90 (30 min)	(Horiuchi et al., 2016)

Legend: Survey of kinetic parameters and enzyme properties from published work and this work. A comparison of kinetic parameters and the effects of pH and temperature on chitinase activities and stabilities as reported in the literature and in this work. CC, colloidal chitinase

2 Supplementary Figures

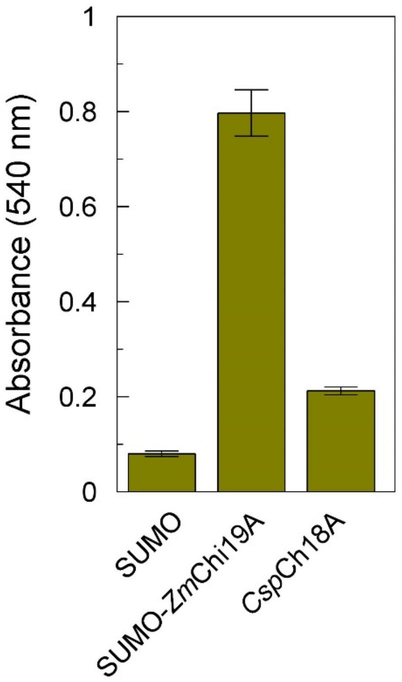

FIGURE S1

Colloidal chitin cleavage activities of *Csp*Chi8A and SUMO-*Zm*Chi18A. The colloidal chitin cleavage activities of *Zea mays* Basic-endochitinase (*Zm*Chi19A) and *Chitinophaga* chitinase sp. (*Csp*Chi18A) are compared with SUMO as a negative control.

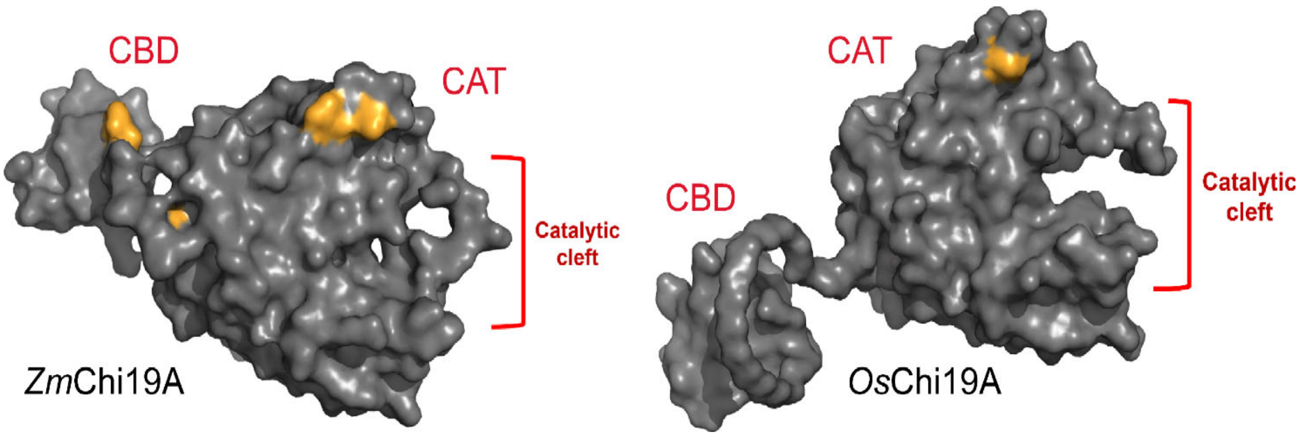

FIGURE S2

Predicted locations of O- glycosylation sites in the 3D structures of full-length enzymes **Left:** *Zea mays* Basic-endochitinase (*Zm*Chi19A) and **Right:** *Oryza sativa* chitinase (*Os*Chi19A). The glycosylation sites were predicted by NetOGlyc version 4, and the images were created using PyMOL version 2.5.2. O-Glycosylation sites shown in orange. *Zm*Chi19A is predicted to have two potential glycosylation sites in the chitin-binding domain, and 2 in the catalytic domain (CAT). *Os*Chi19A is predicted to have one potential glycosylation site in the catalytic domain (CAT). No N-glycosylation sites were predicted by NetNGlyc for either protein.

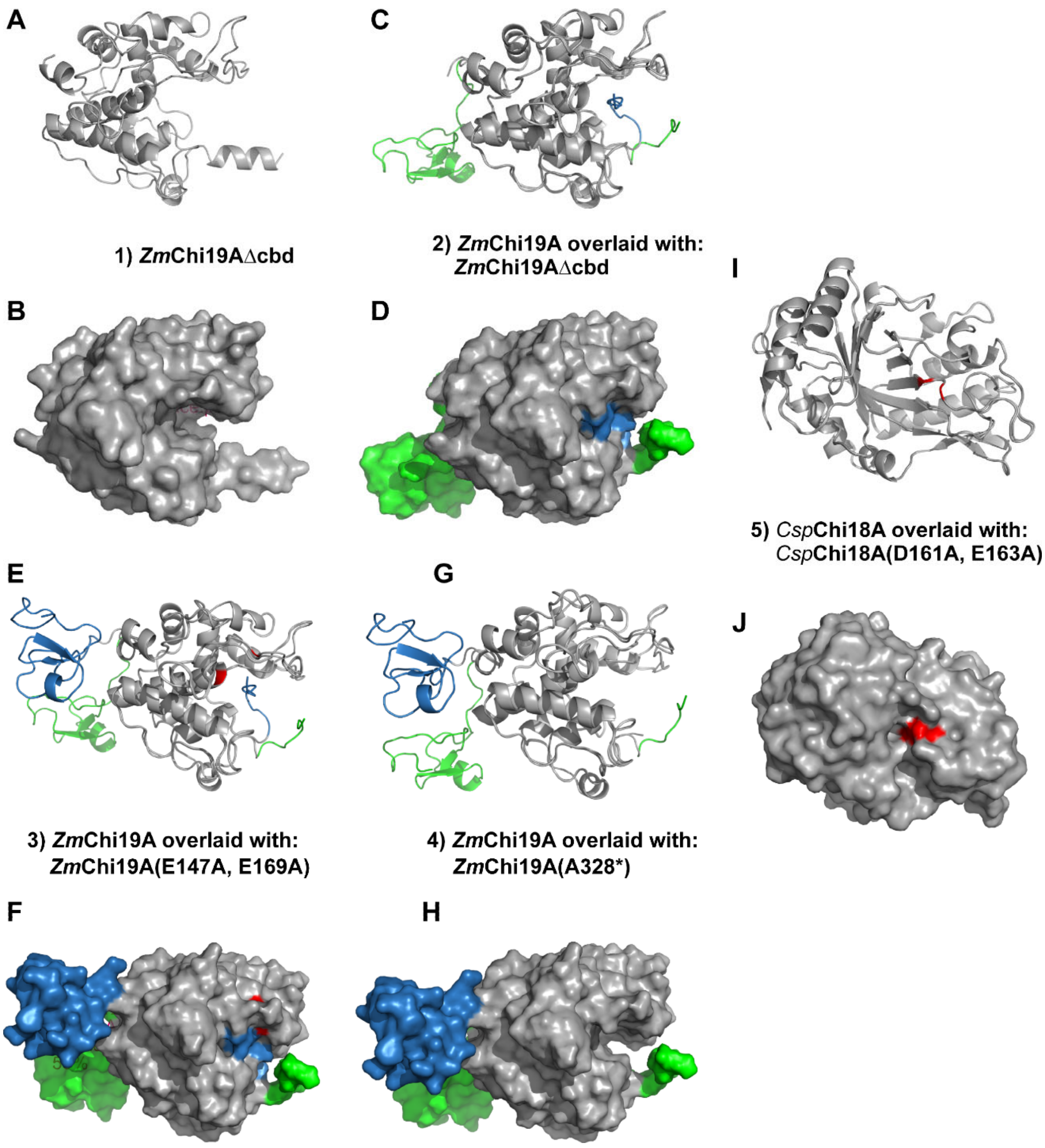

FIGURE S3

Structural alignment of wild-type and mutant chitinases. 1) *Zm*Chi19AΔcbd: A) secondary structure, B) surface structure, 2) Alignment of *Zm*Chi19A with *Zm*Chi19AΔcbd [RMSD = 0.93]: C) aligned secondary structures, D) aligned surface structures. 3) Alignment of *Zm*Chi19A with *Zm*Chi19A(E147A, E169A) [RMSD = 1.32]: E) aligned secondary structures, F) aligned surface structures. 4) Alignment of *Zm*Chi19A with *Zm*Chi19A(A328*) [RMSD = 1.48]: G) aligned secondary structures, H) aligned surface structures. 5) Alignment of *Csp*Chi18A with *Csp*Chi18A(D161A, E163A) [RMSD = 1.17]: I) aligned secondary structures, J) aligned surface structures. Aligned regions shown in grey, unaligned regions shown in green for the native chitinases and blue for the mutants. Mutated amino acids shown in red. The proteins were folded with AlphaFold and mutants were aligned with the native enzymes to evaluate the effects of the mutations on structure.

Supplementary Data

Maize: Zea mays
*Zm*Chi19A   NP_001150560.1, NM_001157088.2   0.542 ROOT QQC___
Atgtcgacaccaggagccccgagcctggccacgacagcggcggctgtcctgtgcgtcctggccgcggcgctcgcggtcac
ggtcgcgagcggccagcagtgcgggcagcaggccggcggcgcaacgtgccgcgactgcctctgctgcagccgcttcggct
tttgcggcgacacctccgagtactgcggcgccgggtgccagagccagtgcaccggctgcggcccgcgtcccgcgggcccc
ggcgtggcatccgtcgtgccgagagacctcttcgagcggctgctcctccaccgcaacgacgcggcgtgccctgcgcgcgg
gttctacacctacgacgcgttcctggccgccgcggccgcgttcccggccttcggcacgacgggcggcgacgagcagcgga
aacgggaggtcgcggcgttcctgggccagacctcccacgagaccacgggcgggtggccgaccgcgcccgacggcccttc
tcgtggggttactgcttcaagcaggagcgcaacccgccgtcggactactgcgagccgcggccgcagtggccgtgcgcgcc
cggcaagaagtacttcggccgcggccccatccagatctccttcaactacaactacggcccggcggggcgagccatcggcg
tggacctcctcaacaacccggacctcgtcgcgacggaccccgtgatatccttcaagacagcgctgtggttctggatgaac
gcgcgggacaacaagccgtcgtgccacgccgtgatcacggggcagtggacgcccacggctgcggacagggcggccggccg
gggcgcgccagggtacggcgtgatcaccaacatcatcaacggtgggatcgagtgcgggcacgggacggacccccgggtca
ccgaccggattggcttctacaagcgctactgcgatgtcttccgcatcggctacgggagcaatctcgattgcgacggccag
aggcccttcaatagcggactggcggttgaggtggcggcgcagtga
MSTPGAPSLATTAAAVLCVLAAALAVTVASGQQCGQQAGGATCRDCLCCSRFGFCGDTSEYCGAGCQSQCTGCGPRPAGP
GVASVVPRDLFERLLLHRNDAACPARGFYTYDAFLAAAAAFPAFGTTGGDEQRKREVAAFLGQTSHETTGGWPTAPDGPF
SWGYCFKQERNPPSDYCEPRPQWPCAPGKKYFGRGPIQISFNYNYGPAGRAIGVDLLNNPDLVATDPVISFKTALWFWMN
ARDNKPSCHAVITGQWTPTAADRAAGRGAPGYGVITNIINGGIECGHGTDPRVTDRIGFYKRYCDVFRIGYGSNLDCDGQ
RPFNSGLAVEVAAQ-
MSTPGAPSLA[10]TTAAAVLCVL[20]AAALAVTVAS[30]GQQCGQQAGG[40]ATCRDCLCCS[50]RFGFCGDTSE[60]YCGAGCQSQC[70]T
GCGPRPAGP[80]GVASVVPRDL[90]FERLLLHRND[100]AACPARGFYT[110]YDAFLAAAAA[120]FPAFGTTGGD[130]EQRKREVAAF[1]
[40]LGQTSHETTG[150]GWPTAPDGPF[160]SWGYCFKQER[170]NPPSDYCEPR[180]PQWPCAPGKK[190]YFGRGPIQIS[200]FNYNYGP
AGR[210]AIGVDLLNNP[220]DLVATDPVIS[230]FKTALWFWMN[240]ARDNKPSCHA[250]VITGQWTPTA[260]ADRAAGRGAP[270]GYG
VITNIIN[280]GGIECGHGTD[290]PRVTDRIGFY[300]KRYCDVFRIG[310]YGSNLDCDGQ[320]RPFNSGLAVE[330]VAAQ
Rice: *Oryza sativa*
GenBank: AAL34318.1, GenBank: L40337.1   0.744 ROOT & LEAF EQC___
Atgaaagccacgacgacagcagtggccctcctcgtggccgccgcggccatggtggcgcaggtagtcgccgagcagtgtgg
ctcgcaggccggtggggcgctgtgcccgaactgcctctgctgcagctcctacggctggtgcggctccacttccgactact
gcggtgacggctgccagagccagtgcgatggctgcggcggcggaggaggtggcggcggcgggggcggcggtggaggtggt
ggtggcggcgcggtggaggcggtcgtgtcgaaggagctcttcgagcagctgctgctccaccgcaacgacgcggcgtgccc
cgccagaggg ttctacacctacaacgccttcgtaacagccgccgccgcgttcccggacttcgccgcgacgggagacgacg
aggcgcgcaagcgtgaggtcgccgcgttcctcgggcagacctcccacgagaccacgggcgggtgggcgaccgcgcccgac
ggcccctactcgtggggctactgcttcaaggaggagatcggcgccacggcctcctactgcgtcgccagcgccgagtggcc
gtgcgccccggacaagaagtacttcggccgcggccccatccaactctcctacaactacaactacgggccggcgggggagg
ccatcggcgaggacctcctgaacaacccggagctggtggcgtcggacccggtggtgtcgttcaagacggcgctgtggttc
tggatgacgccgcagtcgccgaagccgtcgtgccacgacgtgatcacggggcagtggacgccgagctcgggggacatcgc
ggccgggcgggtgccggggtacggcgtgatcaccaacatcatcaacggcgggttggagtgcgggttcggccccgacgacc
gggtggcgaaccggatcggcttctaccagcgctactgcaacgtgctcggcatcggctacgggagcaacctcgactgctac
gaccagaggccgttcaacagcgggttggcggcggtgcagtga
(Codon modified for IDT)
Atgaaagccacgacgacagcagtggccctcctcgtggccgccgcggccatggtggcgcaggtagtcgccgagcagtgtgg
ctcgcaggctggtggtgcgctgtgcccgaactgcttatgctgcagctcctacggctggtgcggttccacttccgactact
gcggtgacggttgccagagccagtgcgatggttgcggcggtggaggaggtggtggcggaggtggcggtggtggaggtggt
ggaggcggtgcggtggaggctgtcgtgtcgaaggagttattcgagcagttattactccaccgcaacgacgcggcgtgccc
cgccagaggtttctacacctacaacgccttcgtaacagccgccgctgcgttcccggacttcgctgcgacgggagacgacg
aggcgcgcaagcgtgaggtcgctgctttcttaggtcagacctcccacgagaccacgggcggttgggcgaccgcgcccgac
ggtccctactcgtggggctactgcttcaaggaggagatcggcgccacggcctcctactgcgtcgccagcgctgagtggcc
gtgcgccccggacaagaagtacttcggccgcggccccatccaattatcctacaactacaactacggtccggcgggtgagg
ccatcggcgaggacctcttaaacaacccggagttagtggcgtcggacccggtggtgtcgttcaagacggcgttatggttc
tggatgacgccgcagtcgccgaagccgtcgtgccacgacgtgatcacgggtcagtggacgccgagctcgggtgacatcgc
ggccgggcgggtgccgggttacggtgtgatcaccaacatcatcaacggcggtttggagtgcggtttcggccccgacgacc

gggtggctaaccggatcggtttctaccagcgctactgcaacgtgttaggcatcggctacggtagcaacttagactgctac
gaccagaggccgttcaacagcggtttagctgctgtgcagtga
MKATTTAVALLVAAAAMVAQVVAEQCGSQAGGALCPNCLCCSSYGWCGSTSDYCGDGCQSQCDGCGGGGGGGGGGGGGGG
GGGAVEAVVSKELFEQLLLHRNDAACPARGFYTYNAFVTAAAAFPDFAATGDDEARKREVAAFLGQTSHETTGGWATAPD
GPYSWGYCFKEEIGATASYCVASAEWPCAPDKKYFGRGPIQLSYNYNYGPAGEAIGEDLLNNPELVASDPVVSFKTALWF
WMTPQSPKPSCHDVITGQWTPSSGDIAAGRVPGYGVITNIINGGLECGFGPDDRVANRIGFYQRYCNVLGIGYGSNLDCY
DQRPFNSGLAAVQ-

MKATTTAVAL 10 LVAAAMVAQ 20 VVAEQCGSQA 30 GGALCPNCLC 40 CSSYGWCGST 50 SDYCGDGCQS 60 QCDGCGGGGG 70 G
GGGGGGGGG 80 GGGAVEAVVS 90 KELFEQLLLH 100 RNDAACPARG 110 FYTYNAFVTA 120 AAAFPDFAAT 130 GDDEARKREV 1
40 AAFLGQTSHE 150 TTGGWATAPD 160 GPYSWGYCFK 170 EEIGATASYC 180 VASAEWPCAP 190 DKKYFGRGPI 200 QLSYNYN
YGP 210 AGEAIGEDLL 220 NNPELVASDP 230 VVSFKTALWF 240 WMTPQSPKPS 250 CHDVITGQWT 260 PSSGDIAAGR 270 VPG
YGVITNI 280 INGGLECGFG 290 PDDRVANRIG 300 FYQRYCNVLG 310 IGYGSNLDCY 320 DQRPFNSGLA 330 AVQ*

Bacteria: Chitinophaga sp. 1303 (2832517…2833428)
GenBank: QJB42510.1, GenBank: CP051204.2 0.706 ROOT & SOIL CKK___
Tcagggcgtatacgttacgctgctgccatagaggatgttggacacaccggagaaatacacatgtttgtcggtgctggtga
ggttgtaccacaggtaaataccatagccgttgtttttggtctgggtggccagccagttggcggtgctttgactggtagcc
tgaatatccacagctgcggggccgaggttggccttggtgaggccggccacattgggcacggaatacgtgccatacatggc
attccagctatagttgataaagtcgcctgctttttttgccgccccaggacaaacgggaagccgccggaccgtaataataga
acgatattattttggtgggcatcagcttccgcagctcatcgaggagcatgacaaaggagctgtcattgggttgtggcagc
ccattgttaccatagtcggcatattcgtcgtcgaaatcaatgccatccagcccgtagtaattggcggtatcggccagctg
ctgggcgaaggctttggcagcagcacggctggtgaagttacagaagccggcaccctgatggtttccaaggatagacaata
atactttcatgcctttgttctgcagcggcacaatctgtgtgtttttgttcaccagcacgttggtcacgttcggattgttg
tacaatacggccttaccggtggtggtgttgtaattgatgttcgctgcgaaaatgatggcgatgtcaaacagttgttgacc
gccggtggtcagggtgtacttaccggtattcaggaggctgttgctgttgacttcaacataacagacagatttaccacctg
ctttggtaacggtttccaccctgggttcagatttggtttgcgggttgtcgggcctggcttcgtctttttttacaggaagcc
acaaacagaagggttgaacaggaggtcagcatatgctgacctcctgttcaacccttctgtttgtggcttcctgtaaaaaa
gacgaagccaggcccgacaacccgcaaaccaaatctgaacccagggtggaaaccgttaccaaagcaggtggtaaatctgt
ctgttatgttgaagtcaacagcaacagcctcctgaataccggtaagtacaccctgaccaccggcggtcaacaactgtttg
acatcgccatcattttcgcagcgaacatcaattacaacaccaccaccggtaaggccgtattgtacaacaatccgaacgtg
accaacgtgctggtgaacaaaaacacacagattgtgccgctgcagaacaaaggcatgaaagtattattgtctatccttgg
aaaccatcagggtgccggcttctgtaacttcaccagccgtgctgctgccaaagccttcgcccagcagctggccgataccg
ccaattactacgggctggatggcattgatttcgacgacgaatatgccgactatggtaacaatgggctgccacaacccaat
gacagctcctttgtcatgctcctcgatgagctgcggaagctgatgcccaccaaaataatatcgttctattattacggtcc
ggcggcttcccgtttgtcctggggcggcaaaaaagcaggcgactttatcaactatagctggaatgccatgtatggcacgt
attccgtgcccaatgtggccggcctcaccaaggccaacctcggccccgcagctgtggatattcaggctaccagtcaaagc
accgccaactggctggccacccagaccaaaaacaacggctatggtatttacctgtggtacaacctcaccagcaccgacaa
acatgtgtatttctccggtgtgtccaacatcctctatggcagcagcgtaacgtatacgccctgA
MLTSCSTLLFVASCKKDEARPDNPQTKSEPRVETVTKAGGKSVCYVEVNSNSLLNTGKYTLTTGGQQLFDIAIIFAANIN
YNTTTGKAVLYNNPNVTNVLVNKNTQIVPLQNKGMKVLLSILGNHQGAGFCNFTSRAAAKAFAQQLADTANYYGLDGIDF
DDEYADYGNNGLPQPNDSSFVMLLDELRKLMPTKIISFYYYGPAASRLSWGGKKAGDFINYSWNAMYGTYSVPNVAGLTK
ANLGPAAVDIQATSQSTANWLATQTKNNGYGIYLWYNLTSTDKHVYFSGVSNILYGSSVTYTP-

MLTSCSTLLF 10 VASCKKDEAR 20 PDNPQTKSEP 30 RVETVTKAGG 40 KSVCYVEVNS 50 NSLLNTGKYT 60 LTTGGQQLFD 70 I
AIIFAANIN 80 YNTTTGKAVL 90 YNNPNVTNVL 100 VNKNTQIVPL 110 QNKGMKVLLS 120 ILGNHQGAGF 130 CNFTSRAAAK 1
40 AFAQQLADTA 150 NYYGLDGIDF 160 DDEYADYGNN 170 GLPQPNDSSF 180 VMLLDELRKL 190 MPTKIISFYY 200 YGPAASR
LSW 210 GGKKAGDFIN 220 YSWNAMYGTY 230 SVPNVAGLTK 240 ANLGPAAVDI 250 QATSQSTANW 260 LATQTKNNGY 270 GIY
LWYNLTS 280 TDKHVYFSGV 290 SNILYGSSVT 300 YTP*

p28b-SUMO-TTBasic-Endochitinase-A: [BC: 138.3.2] 6557 bp (bp = 390: 918) (a.a = 130: 306) [with SUMO-&His-tag = 47.53 kDa, no tag = 32.54 kDa]

KanR

tggcgaatgggacgcgccctgtagcggcgcattaagcgcggcgggtgtggtggttacgcgcagcgtgaccgctacacttg
ccagcgccctagcgcccgctcctttcgctttcttcccttcctttctcgccacgttcgccggctttccccgtcaagctcta
aatcgggggctccctttagggttccgatttagtgctttacggcacctcgaccccaaaaaacttgattagggtgatggttc
acgtagtgggccatcgccctgatagacggtttttcgccctttgacgttggagtccacgttctttaatagtggactcttgt
tccaaactggaacaacactcaaccctatctcggtctattcttttgatttataagggattttgccgatttcggcctattgg
ttaaaaaatgagctgatttaacaaaaatttaacgcgaattttaacaaaatattaacgtttacaatttcaggtggcacttt
tcggggaaatgtgcgcggaacccctatttgtttatttttctaaatacattcaaatatgtatccgctcatgaattaattct
tagaaaaactcatcgagcatcaaatgaaactgcaatttattcatatcaggattatcaataccatatttttgaaaaagccg
tttctgtaatgaaggagaaaactcaccgaggcagttccataggatggcaagatcctggtatcggtctgcgattccgactc
gtccaacatcaatacaacctattaatttcccctcgtcaaaaataaggttatcaagtgagaaatcaccatgagtgacgact
gaatccggtgagaatggcaaaagtttatgcatttctttccagacttgttcaacaggccagccattacgctcgtcatcaaa
atcactcgcatcaaccaaaccgttattcattcgtgattgcgcctgagcgagacgaaatacgcgatcgctgttaaaaggac
aattacaaacaggaatcgaatgcaaccggcgcaggaacactgccagcgcatcaacaatattttcacctgaatcaggatat
tcttctaatacctggaatgctgttttcccggggatcgcagtggtgagtaaccatgcatcatcaggagtacggataaaatg
cttgatggtcggaagaggcataaattccgtcagccagtttagtctgaccatctcatctgtaacatcattggcaacgctac
ctttgccatgtttcagaaacaactctggcgcatcgggcttcccatacaatcgatagattgtcgcacctgattgcccgaca
ttatcgcgagcccatttatacccatataaatcagcatccatgttggaatttaatcgcggcctagagcaagacgtttcccg
ttgaatatggctcataacaccccttgtattactgtttatgtaagcagacagttttattgttcatgaccaaaatcccttaa
cgtgagttttcgttccactgagcgtcagaccccgtagaaaagatcaaaggatcttcttgagatcctttttttctgcgcgt
aatctgctgcttgcaaacaaaaaaaccaccgctaccagcggtggtttgtttgccggatcaagagctaccaactctttttc
cgaaggtaactggcttcagcagagcgcagataccaaatactgtccttctagtgtagccgtagttaggccaccacttcaag
aactctgtagcaccgcctacatacctcgctctgctaatcctgttaccagtggctgctgccagtggcgataagtcgtgtct
taccgggttggactcaagacgatagttaccggataaggcgcagcggtcgggctgaacggggggttcgtgcacacagccca
gcttggagcgaacgacctacaccgaactgagatacctacagcgtgagctatgagaaagcgccacgcttcccgaagggaga
aaggcggacaggtatccggtaagcggcagggtcggaacaggagagcgcacgagggagcttccagggggaaacgcctggta
tctttatagtcctgtcgggtttcgccacctctgacttgagcgtcgatttttgtgatgctcgtcaggggggcggagcctat
ggaaaaacgccagcaacgcggcctttttacggttcctggccttttgctggccttttgctcacatgttctttcctgcgtta
tcccctgattctgtggataaccgtattaccgcctttgagtgagctgataccgctcgccgcagccgaacgaccgagcgcag
cgagtcagtgagcgaggaagcggaagagcgcctgatgcggtattttctccttacgcatctgtgcggtatttcacaccgca
tatatggtgcactctcagtacaatctgctctgatgccgcatagttaagccagtatacactccgctatcgctacgtgactg
ggtcatggctgcgccccgacacccgccaacacccgctgacgcgccctgacgggcttgtctgctcccggcatccgcttaca
gacaagctgtgaccgtctccgggagctgcatgtgtcagaggttttcaccgtcatcaccgaaacgcgcgaggcagctgcgg
taaagctcatcagcgtggtcgtgaagcgattcacagatgtctgcctgttcatccgcgtccagctcgttgagtttctccag
aagcgttaatgtctggcttctgataaagcgggccatgttaagggcggtttttttcctgtttggtcactgatgcctccgtgt
aagggggatttctgttcatgggggtaatgataccgatgaaacgagagaggatgctcacgatacgggttactgatgatgaa
catgcccggttactggaacgttgtgagggtaaacaactggcggtatggatgcggcgggaccagagaaaaatcactcaggg
tcaatgccagcgcttcgttaatacagatgtaggtgttccacagggtagccagcagcatcctgcgatgcagatccggaaca
taatggtgcagggcgctgacttccgcgtttccagactttacgaaacacggaaaccgaagaccattcatgttgttgctcag
gtcgcagacgttttgcagcagcagtcgcttcacgttcgctcgcgtatcggtgattcattctgctaaccagtaaggcaacc
ccgccagcctagccgggtcctcaacgacaggagcacgatcatgcgcacccgtggggccgccatgccggcgataatggcct
gcttctcgccgaaacgtttggtggcgggaccagtgacgaaggcttgagcgagggcgtgcaagattccgaataccgcaagc
gacaggccgatcatcgtcgcgctccagcgaaagcggtcctcgccgaaaatgacccagagcgctgccggcacctgtcctac
gagttgcatgataaagaagacagtcataagtgcggcgacgatagtcatgccccgcgcccaccggaaggagctgactgggt
tgaaggctctcaagggcatcggtcgagatcccggtgcctaatgagtgagctaacttacattaattgcgttgcgctcactg
cccgctttccagtcgggaaacctgtcgtgccagctgcattaatgaatcggccaacgcgcggggagaggcggtttgcgtat
tgggcgccagggtggtttttcttttcaccagtgagacgggcaacagctgattgcccttcaccgcctggccctgagagagt
tgcagcaagcggtccacgctggtttgccccagcaggcgaaaatcctgtttgatggtggttaacggcgggatataacatga

gctgtcttcggtatcgtcgtatcccactaccgagatatccgcaccaacgcgcagcccggactcggtaatggcgcgcattg
cgcccagcgccatctgatcgttggcaaccagcatcgcagtgggaacgatgccctcattcagcatttgcatggtttgttga
aaaccggacatggcactccagtcgccttcccgttccgctatcggctgaatttgattgcgagtgagatatttatgccagcc
agccagacgcagacgcgccgagacagaacttaatgggcccgctaacagcgcgatttgctggtgacccaatgcgaccagat
gctccacgcccagtcgcgtaccgtcttcatgggagaaaataatactgttgatgggtgtctggtcagagacatcaagaaat
aacgccggaacattagtgcaggcagcttccacagcaatggcatcctggtcatccagcggatagttaatgatcagcccact
gacgcgttgcgcgagaagattgtgcaccgccgctttacaggcttcgacgccgcttcgttctaccatcgacaccaccacgc
tggcacccagttgatcggcgcgagatttaatcgccgcgacaatttgcgacggcgcgtgcagggccagactggaggtggca
acgccaatcagcaacgactgtttgcccgccagttgttgtgccacgcggttgggaatgtaattcagctccgccatcgccgc
ttccactttttcccgcgttttcgcagaaacgtggctggcctggttcaccacgcgggaaacggtctgataagagacaccgg
catactctgcgacatcgtataacgttactggtttcacattcaccaccctgaattgactctcttccgggcgctatcatgcc
ataccgcgaaaggttttgcgccattcgatggtgtccgggatctcgacgctctcccttatgcgactcctgcattaggaagc
agcccagtagtaggttgaggccgttgagcaccgccgccgcaaggaatggtgcatgcaaggagatggcgcccaacagtccc
ccggccacggggcctgccaccatacccacgccgaaacaagcgctcatgagcccgaagtggcgagcccgatcttccccatc
ggtgatgtcggcgatataggcgccagcaaccgcacctgtggcgccggtgatgccggccacgatgcgtccggcgtagagga
tcgagatctcgatcccgcgaaattaatacgactcactataggggaattgtgagcggataacaattcccctctagaaataa
ttttgtttaactttaagaaggagatatacc**ATGGGCCATCATCATCATCATCATCATCATCATCACAGCAGCGGCCATAT**
CGAAGGTCGTCATATGGCTAGCATGTCGGACTCAGAAGTCAATCAAGAAGCTAAGCCAGAGGTCAAGCCAGAAGTCAAGC
CTGAGACTCACATCAATTTAAAGGTGTCCGATGGATCTTCAGAGATCTTCTTCAAGATCAAAAAGACCACTCCTTTAAGA
AGGCTGATGGAAGCGTTCGCTAAAAGACAGGGTAAGGAAATGGACTCCTTAAGATTCTTGTACGACGGTATTAGAATTCA
AGCTGATCAGACCCCTGAAGATTTGGACATGGAGGATAACGATATTATTGAGGCTCACAGAGAACAGATTGGTGGATCCG
AGAACCTGTATTTCCAAGGTCAGCAGTGCGGGCAGCAGGCCGGCGGCGCAACGTGCCGCGACTGCCTCTGCTGCAGCCGC
TTCGGCTTTTGCGGCGACACCTCCGAGTACTGCGGCGCCGGGTGCCAGAGCCAGTGCACCGGCTGCGGCCCGCGTCCCGC
GGGCCCCGGCGTGGCATCCGTCGTGCCGAGAGACCTCTTCGAGCGGCTGCTCCTCCACCGCAACGACGCGGCGTGCCCTG
CGCGCGGGTTCTACACCTACGACGCGTTCCTGGCCGCCGCGGCCGCGTTCCCGGCCTTCGGCACGACGGGCGGCGACGAG
CAGCGGAAACGGGAGGTCGCGGCGTTCCTGGGCCAGACCTCCCACGAGACCACGGGCGGGTGGCCGACCGCGCCCGACGG
GCCCTTCTCGTGGGGTTACTGCTTCAAGCAGGAGCGCAACCCGCCGTCGGACTACTGCGAGCCGCGGCCGCAGTGGCCGT
GCGCGCCCGGCAAGAAGTACTTCGGCCGCGGCCCCATCCAGATCTCCTTCAACTACAACTACGGCCCGGCGGGGCGAGCC
ATCGGCGTGGACCTCCTCAACAACCCGGACCTCGTCGCGACGGACCCCGTGATATCCTTCAAGACAGCGCTGTGGTTCTG
GATGAACGCGCGGGACAACAAGCCGTCGTGCCACGCCGTGATCACGGGGCAGTGGACGCCCACGGCTGCGGACAGGGCGG
CCGGCCGGGGCGCGCCAGGGTACGGCGTGATCACCAACATCATCAACGGTGGGATCGAGTGCGGGCACGGGACGGACCCC
CGGGTCACCGACCGGATTGGCTTCTACAAGCGCTACTGCGATGTCTTCCGCATCGGCTACGGGAGCAATCTCGATTGCGA
CGGCCAGAGGCCCTTCAATAGCGGACTGGCGGTTGAGGTGGCGGCGCAGTAATGATAGaaagcttgcggccgcactcgag
caccaccaccaccaccactgagatccggctgctaacaaagcccgaaaggaagctgagttggctgctgccaccgctgagca
ataactagcataaccccttggggcctctaaacgggtcttgaggggttttttgctgaaaggaggaactatatccggat

MGHHHHHHHHHHSSGHIEGRHMASMSDSEVNQEAKPEVKPEVKPETHINLKVSDGSSEIFFKIKKTTPLRRLMEAFAKRQ
GKEMDSLRFLYDGIRIQADQTPEDLDMEDNDIIEAHREQIGGSENLYFQGQQCGQQAGGATCRDCLCCSRFGFCGDTSEY
CGAGCQSQCTGCGPRPAGPGVASVVPRDLFERLLLHRNDAACPARGFYTYDAFLAAAAAFPAFGTTGGDEQRKREVAAFL
GQTSHETTGGWPTAPDGPFSWGYCFKQERNPPSDYCEPRPQWPCAPGKKYFGRGPIQISFNYNYGPAGRAIGVDLLNNPD
LVATDPVISFKTALWFWMNARDNKPSCHAVITGQWTPTAADRAAGRGAPGYGVITNIINGGIECGHGTDPRVTDRIGFYK
RYCDVFRIGYGSNLDCDGQRPFNSGLAVEVAAQ***

Original pET28b-10xHis-SUMO vector: 5636 bp
[BC: 139.8.1] (bp = 444) (a.a = 148)
[with His-tag = 16.84 kDa]
KanR

```
tggcgaatgggacgcgccctgtagcggcgcattaagcgcggcgggtgtggtggttacgcgcagcgtgaccgctacacttg
ccagcgccctagcgcccgctcctttcgctttcttcccttcctttctcgccacgttcgccggctttccccgtcaagctcta
aatcgggggctccctttagggttccgatttagtgctttacggcacctcgaccccaaaaaacttgattagggtgatggttc
acgtagtgggccatcgccctgatagacggtttttcgccctttgacgttggagtccacgttctttaatagtggactcttgt
tccaaactggaacaacactcaaccctatctcggtctattcttttgatttataagggattttgccgatttcggcctattgg
ttaaaaaatgagctgatttaacaaaaatttaacgcgaattttaacaaaatattaacgtttacaatttcaggtggcacttt
tcggggaaatgtgcgcggaacccctatttgtttatttttctaaatacattcaaatatgtatccgctcatgaattaattct
tagaaaaactcatcgagcatcaaatgaaactgcaatttattcatatcaggattatcaataccatatttttgaaaaagccg
tttctgtaatgaaggagaaaactcaccgaggcagttccataggatggcaagatcctggtatcggtctgcgattccgactc
gtccaacatcaatacaacctattaatttcccctcgtcaaaaataaggttatcaagtgagaaatcaccatgagtgacgact
gaatccggtgagaatggcaaaagtttatgcatttctttccagacttgttcaacaggccagccattacgctcgtcatcaaa
atcactcgcatcaaccaaaccgttattcattcgtgattgcgcctgagcgagacgaaatacgcgatcgctgttaaaaggac
aattacaaacaggaatcgaatgcaaccggcgcaggaacactgccagcgcatcaacaatattttcacctgaatcaggatat
tcttctaatacctggaatgctgttttcccggggatcgcagtggtgagtaaccatgcatcatcaggagtacggataaaatg
cttgatggtcggaagaggcataaattccgtcagccagtttagtctgaccatctcatctgtaacatcattggcaacgctac
ctttgccatgtttcagaaacaactctggcgcatcgggcttcccatacaatcgatagattgtcgcacctgattgcccgaca
ttatcgcgagcccatttatacccatataaatcagcatccatgttggaatttaatcgcggcctagagcaagacgtttcccg
ttgaatatggctcataacaccccttgtattactgtttatgtaagcagacagttttattgttcatgaccaaaatcccttaa
cgtgagttttcgttccactgagcgtcagaccccgtagaaaagatcaaaggatcttcttgagatcctttttttctgcgcgt
aatctgctgcttgcaaacaaaaaaaccaccgctaccagcggtggtttgtttgccggatcaagagctaccaactctttttc
cgaaggtaactggcttcagcagagcgcagataccaaatactgtccttctagtgtagccgtagttaggccaccacttcaag
aactctgtagcaccgcctacatacctcgctctgctaatcctgttaccagtggctgctgccagtggcgataagtcgtgtct
taccgggttggactcaagacgatagttaccggataaggcgcagcggtcgggctgaacggggggttcgtgcacacagccca
gcttggagcgaacgacctacaccgaactgagatacctacagcgtgagctatgagaaagcgccacgcttcccgaagggaga
aaggcggacaggtatccggtaagcggcagggtcggaacaggagagcgcacgagggagcttccagggggaaacgcctggta
tctttatagtcctgtcgggtttcgccacctctgacttgagcgtcgatttttgtgatgctcgtcaggggggcggagcctat
ggaaaaacgccagcaacgcggcctttttacggttcctggccttttgctggccttttgctcacatgttctttcctgcgtta
tcccctgattctgtggataaccgtattaccgcctttgagtgagctgataccgctcgccgcagccgaacgaccgagcgcag
cgagtcagtgagcgaggaagcggaagagcgcctgatgcggtattttctccttacgcatctgtgcggtatttcacaccgca
tatatggtgcactctcagtacaatctgctctgatgccgcatagttaagccagtatacactccgctatcgctacgtgactg
ggtcatggctgcgccccgacacccgccaacacccgctgacgcgccctgacgggcttgtctgctcccggcatccgcttaca
gacaagctgtgaccgtctccgggagctgcatgtgtcagaggttttcaccgtcatcaccgaaacgcgcgaggcagctgcgg
taaagctcatcagcgtggtcgtgaagcgattcacagatgtctgcctgttcatccgcgtccagctcgttgagtttctccag
aagcgttaatgtctggcttctgataaagcgggccatgttaagggcggttttttcctgtttggtcactgatgcctccgtgt
aagggggatttctgttcatgggggtaatgataccgatgaaacgagagaggatgctcacgatacgggttactgatgatgaa
catgcccggttactggaacgttgtgagggtaaacaactggcggtatggatgcggcgggaccagagaaaaatcactcaggg
tcaatgccagcgcttcgttaatacagatgtaggtgttccacagggtagccagcagcatcctgcgatgcagatccggaaca
taatggtgcagggcgctgacttccgcgtttccagactttacgaaacacggaaaccgaagaccattcatgttgttgctcag
gtcgcagacgttttgcagcagcagtcgcttcacgttcgctcgcgtatcggtgattcattctgctaaccagtaaggcaacc
ccgccagcctagccgggtcctcaacgacaggagcacgatcatgcgcacccgtggggccgccatgccggcgataatggcct
gcttctcgccgaaacgtttggtggcgggaccagtgacgaaggcttgagcgagggcgtgcaagattccgaataccgcaagc
gacaggccgatcatcgtcgcgctccagcgaaagcggtcctcgccgaaaatgacccagagcgctgccggcacctgtcctac
gagttgcatgataaagaagacagtcataagtgcggcgacgatagtcatgccccgcgcccaccggaaggagctgactgggt
tgaaggctctcaagggcatcggtcgagatcccggtgcctaatgagtgagctaacttacattaattgcgttgcgctcactg
cccgctttccagtcgggaaacctgtcgtgccagctgcattaatgaatcggccaacgcgcggggagaggcggtttgcgtat
tgggcgccagggtggtttttcttttcaccagtgagacgggcaacagctgattgcccttcaccgcctggccctgagagagt
tgcagcaagcggtccacgctggtttgccccagcaggcgaaaatcctgtttgatggtggttaacggcgggatataacatga
gctgtcttcggtatcgtcgtatcccactaccgagatatccgcaccaacgcgcagcccggactcggtaatggcgcgcattg
cgcccagcgccatctgatcgttggcaaccagcatcgcagtgggaacgatgccctcattcagcatttgcatggtttgttga
aaaccggacatggcactccagtcgccttcccgttccgctatcggctgaatttgattgcgagtgagatatttatgccagcc
agccagacgcagacgcgccgagacagaacttaatgggcccgctaacagcgcgatttgctggtgacccaatgcgaccagat
gctccacgcccagtcgcgtaccgtcttcatgggagaaaataatactgttgatgggtgtctggtcagagacatcaagaaat
aacgccggaacattagtgcaggcagcttccacagcaatggcatcctggtcatccagcggatagttaatgatcagcccact
```

gacgcgttgcgcgagaagattgtgcaccgccgctttacaggcttcgacgccgcttcgttctaccatcgacaccaccacgc
tggcacccagttgatcggcgcgagatttaatcgccgcgacaatttgcgacggcgcgtgcagggccagactggaggtggca
acgccaatcagcaacgactgtttgcccgccagttgttgtgccacgcggttgggaatgtaattcagctccgccatcgccgc
ttccactttttcccgcgttttcgcagaaacgtggctggcctggttcaccacgcgggaaacggtctgataagagacaccgg
catactctgcgacatcgtataacgttactggtttcacattcaccaccctgaattgactctcttccgggcgctatcatgcc
ataccgcgaaaggttttgcgccattcgatggtgtccgggatctcgacgctctcccttatgcgactcctgcattaggaagc
agcccagtagtaggttgaggccgttgagcaccgccgccgcaaggaatggtgcatgcaaggagatggcgcccaacagtccc
ccggccacggggcctgccaccatacccacgccgaaacaagcgctcatgagcccgaagtggcgagcccgatcttccccatc
ggtgatgtcggcgatataggcgccagcaaccgcacctgtggcgccggtgatgccggccacgatgcgtccggcgtagagga
tcgagatctcgatcccgcgaaattaatacgactcactataggggaattgtgagcggataacaattcccctctagaaataa
ttttgtttaactttaagaaggagatatacc**ATGGGCCATCATCATCATCATCATCATCATCATCACAGCAGCGGCCATAT**
CGAAGGTCGTCATATGGCTAGCATGTCGGACTCAGAAGTCAATCAAGAAGCTAAGCCAGAGGTCAAGCCAGAAGTCAAGC
CTGAGACTCACATCAATTTAAAGGTGTCCGATGGATCTTCAGAGATCTTCTTCAAGATCAAAAAGACCACTCCTTTAAGA
AGGCTGATGGAAGCGTTCGCTAAAAGACAGGGTAAGGAAATGGACTCCTTAAGATTCTTGTACGACGGTATTAGAATTCA
AGCTGATCAGACCCCTGAAGATTTGGACATGGAGGATAACGATATTATTGAGGCTCACAGAGAACAGATTGGTGGATCCG
AATTCGAGCTCCGTCGACAAGCTTGCGGCCGCACTCGAGCACCACCACCACCACCACTGAGATCCGGCTGCTAAcaaagc
ccgaaaggaagctgagttggctgctgccaccgctgagcaataactagcataaccccttggggcctctaaacgggtcttga
ggggttttttgctgaaaggaggaactatatccggat

MGHHHHHHHHHHSSGHIEGRHMASMSDSEVNQEAKPEVKPEVKPETHINLKVSDGSSEIFFKIKKTTPLRRLMEAFAKRQ
GKEMDSLRFLYDGIRIQADQTPEDLDMEDNDIIEAHREQIGGSEFELRRQACGRTRAPPPPPLRSGC*

Nucleotide Sequence (Wild-type):
ATTGGTGGATCCGAGAACCTGTACTTTCAGGGCCAGCAGTGCGGGCAGCAGGCCGGCGGCGCAACGTGCCGCGACTGCCT
CTGCTGCAGCCGCTTCGGCTTTTGCGGCGACACCTCCGAGTACTGCGGCGCCGGGTGCCAGAGCCAGTGCACCGGCTGCG
GCCCGCGTCCCGCGGGCCCCGGCGTGGCATCCGTCGTGCCGAGAGACCTCTTCGAGCGGCTGCTCCTCCACCGCAACGAC
GCGGCGTGCCCTGCGCGCGGGTTCTACACCTACGACGCGTTCCTGGCCGCCGCGGCCGCGTTCCCGGCCTTCGGCACGAC
GGGCGGCGACGAGCAGCGGAAACGGGAGGTCGCGGCGTTCCTGGGCCAGACCTCCCACGAGACCACGGGCGGGTGGCCGA
CCGCGCCCGACGGGCCCTTCTCGTGGGGTTACTGCTTCAAGCAGGAGCGCAACCCGCCGTCGGACTACTGCGAGCCGCGG
CCGCAGTGGCCGTGCGCGCCCGGCAAGAAGTACTTCGGCCGCGGCCCCATCCAGATCTCCTTCAACTACAACTACGGCCC
GGCGGGGCGAGCCATCGGCGTGGACCTCCTCAACAACCCGGACCTCGTCGCGACGGACCCCGTGATATCCTTCAAGACAG
CGCTGTGGTTCTGGATGAACGCGCGGGACAACAAGCCGTCGTGCCACGCCGTGATCACGGGGCAGTGGACGCCCACGGCT
GCGGACAGGGCGGCCGGCCGGGGCGCGCCAGGGTACGGCGTGATCACCAACATCATCAACGGTGGGATCGAGTGCGGGCA
CGGGACGGACCCCCGGGTCACCGACCGGATTGGCTTCTACAAGCGCTACTGCGATGTCTTCCGCATCGGCTACGGGAGCA
ATCTCGATTGCGACGGCCAGAGGCCCTTCAATAGCGGACTGGCGGTTGAGGTGGCGGCGCAGTAATGATAGAAAGCTTGC
GGCC

Nucleotide Sequence (M1 + M2):
ATTGGTGGATCCGAGAACCTGTACTTTCAGGGCCAGCAGTGCGGGCAGCAGGCCGGCGGCGCAACGTGCCGCGACTGCCT
CTGCTGCAGCCGCTTCGGCTTTTGCGGCGACACCTCCGAGTACTGCGGCGCCGGGTGCCAGAGCCAGTGCACCGGCTGCG
GCCCGCGTCCCGCGGGCCCCGGCGTGGCATCCGTCGTGCCGAGAGACCTCTTCGAGCGGCTGCTCCTCCACCGCAACGAC
GCGGCGTGCCCTGCGCGCGGGTTCTACACCTACGACGCGTTCCTGGCCGCCGCGGCCGCGTTCCCGGCCTTCGGCACGAC
GGGCGGCGACGAGCAGCGGAAACGGGAGGTCGCGGCGTTCCTGGGCCAGACCTCCCACGCGACCACGGGCGGGTGGCCGA
CCGCGCCCGACGGGCCCTTCTCGTGGGGTTACTGCTTCAAGCAGGCGCGCAACCCGCCGTCGGACTACTGCGAGCCGCGG
CCGCAGTGGCCGTGCGCGCCCGGCAAGAAGTACTTCGGCCGCGGCCCCATCCAGATCTCCTTCAACTACAACTACGGCCC
GGCGGGGCGAGCCATCGGCGTGGACCTCCTCAACAACCCGGACCTCGTCGCGACGGACCCCGTGATATCCTTCAAGACAG
CGCTGTGGTTCTGGATGAACGCGCGGGACAACAAGCCGTCGTGCCACGCCGTGATCACGGGGCAGTGGACGCCCACGGCT
GCGGACAGGGCGGCCGGCCGGGGCGCGCCAGGGTACGGCGTGATCACCAACATCATCAACGGTGGGATCGAGTGCGGGCA
CGGGACGGACCCCCGGGTCACCGACCGGATTGGCTTCTACAAGCGCTACTGCGATGTCTTCCGCATCGGCTACGGGAGCA
ATCTCGATTGCGACGGCCAGAGGCCCTTCAATAGCGGACTGGCGGTTGAGGTGGCGGCGCAGTAATGATAGAAAGCTTGC
GGCC

G-block Nucleotide Sequence (M1 + M2_IDT Codon modified):
ATTGGTGGATCCGAGAACCTGTACTTTCAGGGCCAGCAGTGCGGGCAGCAGGCAGGCGGTGCGACGTGCAGAGACTGCCT
GTGTTGTTCTAGATTTGGCTTTTGTGGGGACACTTCGGAGTACTGTGGTGCCGGTTGTCAGAGTCAATGTACCGGTTGTG
GTCCCCGCCCTGCAGGTCCGGGTGTCGCGTCCGTTGTTCCAAGAGATTTGTTCGAGCGTCTTTTATTACACCGTAACGAC
GCCGCGTGCCCAGCAAGAGGGTTTTATACGTATGACGCGTTCCTTGCAGCGGCCGCCGCTTTCCCAGCCTTCGGGACTAC
GGGAGGAGACGAGCAGCGCAAAAGAGAGGTTGCTGCTTTTTTAGGCCAGACATCTCACGCGACAACGGGCGGGTGGCCAA
CGGCACCCGATGGACCCTTTAGCTGGGGATACTGTTTTAAGCAGGCGCGTAATCCACCATCAGACTATTGTGAGCCAAGA
CCACAGTGGCCTTGCGCCCCTGGCAAAAAATATTTTGGTCGCGGTCCGATACAAATTTCGTTTAACTACAACTATGGCCC
AGCCGGCCGTGCTATTGGTGTAGACCTTTTAAATAATCCAGACCTGGTTGCTACTGACCCAGTGATTAGTTTTAAAACTG
CGCTTTGGTTCTGGATGAATGCTCGTGATAATAAACCGAGCTGTCATGCGGTCATCACGGGACAGTGGACTCCAACCGCC
GCGGATCGTGCGGCGGGACGGGGAGCACCGGGGTATGGTGTCATAACGAACATTATAAACGGGGGAATAGAGTGCGGGCA
TGGGACTGACCCGCGTGTAACGGACCGGATAGGCTTTTACAAACGCTACTGTGACGTGTTTCGTATAGGTTATGGAAGCA
ACCTTGATTGTGATGGGCAACGTCCCTTCAATAGCGGACTGGCGGTTGAGGTGGCGGCGCAGTAATGATAGAAAGCTTGC
GGCC

Cloned Nucleotide Sequence:
ATGAGAGGATCGCATCACCATCACCATCACGGATCCATGTGTAAAAAAGACGAAGCCAGGCCCGACAACCCGCAAACCAAA
TCTGAACCCAGGGTGGAAACCGTTACCAAAGCAGGTGGTAAATCTGTCTGTTATGTTGAAGTCAACAGCAACAGCCTCCTG
AATACCGGTAAGTACACCCTGACCACCGGCGGTCAACAACTGTTTGACATCGCCATCATTTTCGCAGCGAACATCAATTAC
AACACCACCACCGGTAAGGCCGTATTGTACAACAATCCGAACGTGACCAACGTGCTGGTGAACAAAAACACACAGATTGTG
CCGCTGCAGAACAAAGGCATGAAAGTATTATTGTCTATCCTTGGAAACCATCAGGGTGCCGGCTTCTGTAACTTCACCAGC
CGTGCTGCTGCCAAAGCCTTCGCCCAGCAGCTGGCCGATACCGCCAATTACTACGGGCTGGATGGCATTGATTTCGACGAC
GAATATGCCGACTATGGTAACAATGGGCTGCCACAACCCAATGACAGCTCCTTTGTCATGCTCCTCGATGAGCTGCGGAAG
CTGATGCCCACCAAAATAATATCGTTCTATTATTACGGTCCGGCGGCTTCCCGTTTGTCCTGGGGCGGCAAAAAAGCAGGC
GACTTTATCAACTATAGCTGGAATGCCATGTATGGCACGTATTCCGTGCCCAATGTGGCCGGCCTCACCAAGGCCAACCTC
GGCCCCGCAGCTGTGGATATTCAGGCTACCAGTCAAAGCACCGCCAACTGGCTGGCCACCCAGACCAAAAACAACGGCTAT
GGTATTTACCTGTGGTACAACCTCACCAGCACCGACAAACATGTGTATTTCTCCGGTGTGTCCAACATCCTCTATGGCAGC
AGCGTAACGTATACGCCCTAA

References Cited

Hollis, T., Honda, Y., Fukamizo, T., Marcotte, E., Day, P. J. & Robertus, J. D. (1997). Kinetic analysis of barley chitinase. *Arch Biochem Biophys,* 344 (2)**,** 335-42, doi: 10.1006/abbi.1997.0225

Honda, Y., Kitaoka, M., Tokuyasu, K., Sasaki, C., Fukamizo, T. & Hayashi, K. (2003). Kinetic studies on the hydrolysis of N-acetylated and N-deacetylated derivatives of 4-methylumbelliferyl chitobioside by the family 18 chitinases ChiA and ChiB from Serratia marcescens. *J Biochem,* 133 (2)**,** 253-8, doi: 10.1093/jb/mvg031

Horiuchi, A., Aslam, M., Kanai, T. & Atomi, H. (2016). A Structurally Novel Chitinase from the Chitin-Degrading Hyperthermophilic Archaeon Thermococcus chitonophagus. *Appl Environ Microbiol,* 82 (12)**,** 3554-3562, doi: 10.1128/aem.00319-16

Patel, A. K., Singh, V. K., Yadav, R. P., Moir, A. J. & Jagannadham, M. V. (2009). ICChI, a glycosylated chitinase from the latex of Ipomoea carnea. *Phytochemistry,* 70 (10)**,** 1210-6, doi: 10.1016/j.phytochem.2009.07.005

Rajninec, M., Jopcik, M., Danchenko, M. & Libantova, J. (2020). Biochemical and antifungal characteristics of recombinant class I chitinase from Drosera rotundifolia. *Int J Biol Macromol,* 161**,** 854-863, doi: 10.1016/j.ijbiomac.2020.06.123

Sierra-Gómez, Y., Rodríguez-Hernández, A., Cano-Sánchez, P., Gómez-Velasco, H., Hernández-Santoyo, A., Siliqi, D. & Rodríguez-Romero, A. (2019). A biophysical and structural study of two chitinases from Agave tequilana and their potential role as defense proteins. *The FEBS Journal,* 286 (23)**,** 4778-4796, doi: https://doi.org/10.1111/febs.14993

Sukprasirt, P. & Wititsuwannakul, R. (2014). A chitinolytic endochitinase and β-N-acetylglucosaminidase-based system from Hevea latex in generating N-acetylglucosamine from chitin. *Phytochemistry,* 104**,** 5-11, doi: 10.1016/j.phytochem.2014.04.001

Thimoteo, S. S., Glogauer, A., Faoro, H., De Souza, E. M., Huergo, L. F., Moerschbacher, B. M. & Pedrosa, F. O. (2017). A broad pH range and processive chitinase from a metagenome library. *Braz J Med Biol Res,* 50 (1)**,** e5658, doi: 10.1590/1414-431x20165658

Van Munster, J. M., Sanders, P., Ten Kate, G. A., Dijkhuizen, L. & Van Der Maarel, M. J. (2015). Kinetic characterization of Aspergillus niger chitinase CfcI using a HPAEC-PAD method for native chitin oligosaccharides. *Carbohydr Res,* 407**,** 73-8, doi: 10.1016/j.carres.2015.01.014

Wang, Y. J., Jiang, W. X., Zhang, Y. S., Cao, H. Y., Zhang, Y., Chen, X. L., Li, C. Y., Wang, P., Zhang, Y. Z., Song, X. Y. & Li, P. Y. (2019). Structural Insight Into Chitin Degradation and Thermostability of a Novel Endochitinase From the Glycoside Hydrolase Family 18. *Front Microbiol,* 10**,** 2457, doi: 10.3389/fmicb.2019.02457
